# Direct Atomic‐Level Imaging of Zeolites: Oxygen, Sodium in Na‐LTA and Iron in Fe‐MFI

**DOI:** 10.1002/anie.202006122

**Published:** 2020-07-20

**Authors:** Alvaro Mayoral, Qing Zhang, Yi Zhou, Pengyu Chen, Yanhang Ma, Taro Monji, Pit Losch, Wolfgang Schmidt, Ferdi Schüth, Hajime Hirao, Jihong Yu, Osamu Terasaki

**Affiliations:** ^1^ Centre for High-resolution Electron Microscopy (*C*ħ*EM)* School of Physical Science and Technology ShanghaiTech University 393 Middle Huaxia Road Pudong Shanghai 201210 China; ^2^ Key Laboratory of Biomedical Polymers-Ministry of Education College of Chemistry and Molecular Sciences Wuhan University Wuhan 430072 China; ^3^ Zhiyuan College & School of Chemistry and Chemical Engineering Shanghai Jiao Tong University 800 Dongchuan Road Shanghai 200240 China; ^4^ Hitachi Solutions East (Japan) Ltd. Sendai Japan; ^5^ Department of Heterogeneous Catalysis Max-Planck-Institut für Kohlenforschung Kaiser-Wilhelm-Platz 1 45470 Mülheim an der Ruhr Germany; ^6^ Department of Chemistry City University of Hong Kong Tat Chee Avenue Kowloon, Hong Kong SAR China; ^7^ State Key Laboratory of Inorganic Synthesis and Preparative Chemistry College of Chemistry, International Center of Future Science Jilin University Changchun 130012 China; ^8^ Department of Materials and Environmental Chemistry Stockholm University Stockholm Sweden; ^9^ Institute of Materials Science of Aragon (ICMA), Spanish National Research Council (CSIC) Advanced Microscopy Laboratory (LMA) University of Zaragoza 12, Calle de Pedro Cerbuna 50009 Zaragoza Spain

**Keywords:** annular bright-field analysis, beam damage, electron diffraction, electron microscopy, zeolites

## Abstract

Zeolites are becoming more versatile in their chemical functions through rational design of their frameworks. Therefore, direct imaging of all atoms at the atomic scale, basic units (Si, Al, and O), heteroatoms in the framework, and extra‐framework cations, is needed. TEM provides local information at the atomic level, but the serious problem of electron‐beam damage needs to be overcome. Herein, all framework atoms, including oxygen and most of the extra‐framework Na cations, are successfully observed in one of the most electron‐beam‐sensitive and lowest framework density zeolites, Na‐**LTA**. Zeolite performance, for instance in catalysis, is highly dependent on the location of incorporated heteroatoms. Fe single atomic sites in the **MFI** framework have been imaged for the first time. The approach presented here, combining image analysis, electron diffraction, and DFT calculations, can provide essential structural keys for tuning catalytically active sites at the atomic level.

## Graphical abstract

**Down to the atom**: Through different imaging methods, electron microscopy can provide direct observation of oxygen atoms and sodium cations, pointing the way for the analysis of the local structure around heteroatoms in zeolite frameworks. The data provide clear evidence of Fe on tetrahedral sites within the **MFI** zeolite structure, clearly distinguished as single atoms.

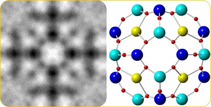

## Introduction

Zeolites are formed by TO_4_ units (T, Si or Al‐atoms) tetrahedrally coordinated through O bridges, and they can generally be described as M^*m*+^_*x*/*m*_[Si_1−*x*_A1_*x*_O_2_], where M is an exchangeable counter cation with valence m^+^ to balance the negative framework charge, and the range of *x* is equal to or less than 0.5 and can be as low as 0.0 for pure silica polymorphs. Historically, zeolite science ranged from natural to synthetic materials, in terms of composition from Si/Al=1 (*x*=0.5) to Si/Al=∞ (*x*=0.0), and according to the nature of the T‐elements, has been extended from Si and Al to P, B, Ge and some transition metals. Functions of zeolites have been widely discussed as a function of structural geometry (sizes of cavities/channels, pore‐opening and connectivity/ dimensionality), composition (especially Si/Al ratio) of the framework, distribution of Al or transition metals and vacancies in the framework.

Using powder or single‐crystal X‐ray diffraction (XRD), most previous work has focused on determining crystal structures of novel zeolites averaged over a large volume, often including occupation probabilities as refined parameters. Electron diffraction (ED) patterns give structural information averaged over the electron‐irradiated volume, which is around few hundred of nanometers, onto a unit cell in momentum space, while electron microscopy (EM) images give pinpoint structural information in real space, although this information is still averaged over the crystal thickness along the electron beam (e‐beam) direction.

For a long time, zeolite structure analysis through HRTEM imaging has been restricted to the observation of pores (not at the atomic level) in terms of size, shape, and their periodic arrangement in the crystals. The limitations mainly originated from the spatial resolution of EM and from the e‐beam damage (highly associated to the Si/Al ratio).^1^

Electron diffraction is not restricted to the same extent by e‐beam sensitivity, as the electron dose is tenths of magnitude lower than in high‐resolution imaging, allowing the solution of very complicated novel zeolite structures (**SFE**), which was solved solely from a set of ED patterns.^2^ However, many basic zeolite framework structures have been determined using 3D ED data by assuming single scattering of electrons.^3^

With the implementation of spherical aberration (*C_s_*) correctors in both scanning/transmission electron microscopes, (S)TEM,^4^ sub‐Ångstrom lateral atomic resolution has been achieved for e‐beam stable inorganic materials.^5^ To date, most results have been acquired using annular dark field (ADF) or high‐angle annular dark field (HAADF) detectors, which are sensitive to the atomic number of the components (Z‐contrast), as the appearance of artefacts in the images is strongly reduced compared to TEM. Alternatively, annular bright field (ABF) can simultaneously provide complementary information on light elements,^6^ a technique that is equivalent to the hollow cone illumination imaging approach.^7^ According to these studies, the contrast transfer is expected to give better resolution than conventional BF and HAADF STEM. The major drawbacks of this approach to be applied over beam sensitive materials are the higher susceptibility to aberrations in the microscope that affect the potential higher resolution, the low signal‐to‐noise ratio as a result of the low‐dose conditions and small collection area and the larger influence on the image of small focus variations.6a

One motivation for applying this technique to zeolites relates to their widespread use as supports for metallic species either in their pores or on their external surfaces. Metal nanoclusters of high atomic number (such as Au, Ir, or Pt) within zeolites have been successfully imaged through HRSTEM; however, location of the clusters and determination of their sizes were not clear, since T‐atoms of the framework were not resolved.^8^ A good example of the minimum requirements for the next generation of functional porous materials concerning the analysis of all T‐atoms (Al and P) in an e‐beam sensitive AlPO_4_ (STA‐20), which were directly observed by *C_s_*‐corrected STEM‐ADF imaging.^9^

In the present work, we demonstrate that two fundamental challenges in the direct atomic‐level imaging of zeolites have now been solved: First, observing all framework atoms, including O‐atoms, in one of the most e‐beam sensitive and lowest framework density zeolites, Na‐**LTA** (with Si/Al≈1). For this analysis, an annular bright field detector (ABF) was employed for the first time in the study of zeolites. To corroborate the feasibility of this approach, silicalite‐1 (**MFI**‐type) was also imaged clearly observing the O‐atoms. Direct observation of O‐atoms is very important, since their positions are very sensitive to the presence of cationic species such as H^+^, which play a crucial role in the catalytic performance of zeolites. Second, direct observation of single transition‐metal (Fe) heteroatoms in silicalite‐**MFI** zeolite, showing clear evidence of a non‐periodic distribution of Fe within the framework. **MFI** has one of the highest framework density and is thermally and chemically very stable; thus, it is one of the most extensively used zeolites in academia and industry. Replacement of Si by transition metal heteroatoms provides enhanced catalytic activity. **MFI** isomorphously substituted with iron, for instance, is active in the selective oxidation of methane to methanol, with the extraction of iron from framework positions to extra‐framework positions yielding the active iron species.^10^ Framework iron species in Fe‐**MFI** were shown to be much more active than extra‐framework species for dehydrogenation of propane.^11^ Considering that Fe has much smaller Z‐number than the traditional noble metals studied by EM, this study is the first direct observation of single transition‐metal atoms in a zeolite framework.

Furthermore, the sensitivity of ED for analysis of the local structure of Na‐**LTA** and Fe‐**MFI** is discussed, and comparison of the experimental data with DFT calculations is reported.

## Results and Discussion

### Direct Observation of the Na‐LTA and Fe‐MFI Structures

The structure of Na‐**LTA** (ideal formula Na_96_Si_96_Al_96_O_384_) determined from single‐crystal XRD by Pluth and Smith is well accepted.^12^ Based on the space group *Fm* $\bar{3}$ *c* (no. 226) with lattice parameter *a*=24.555 Å, the structure was refined by introducing occupation probabilities (OPs) for Na^+^ cations: 62 Na(1) in a 64‐fold position (along 3‐fold axes at the center of single 6‐rings (S6Rs)) with OP=0.972; 23 Na(2) in a 96‐fold position with OP=0.240 inside single 8‐rings (S8Rs), and 6 Na(3) in a 96‐fold position with OP=0.066 close to the double 4‐rings (D4Rs). The authors claimed that Al and Si atoms strictly alternate throughout the framework. However, the Si/Al ratio was slightly greater than unity and five reflections violating the systematic absences of the space group were observed. Such a well‐studied material is therefore a very good candidate for further characterization, not only because deep studies were already performed, but also due to its importance in industry.^13^ This zeolite is one of the most e‐beam sensitive, as Si/Al=1 and the framework density is 12.9/1000 Å^3^.

**Figure 1:**
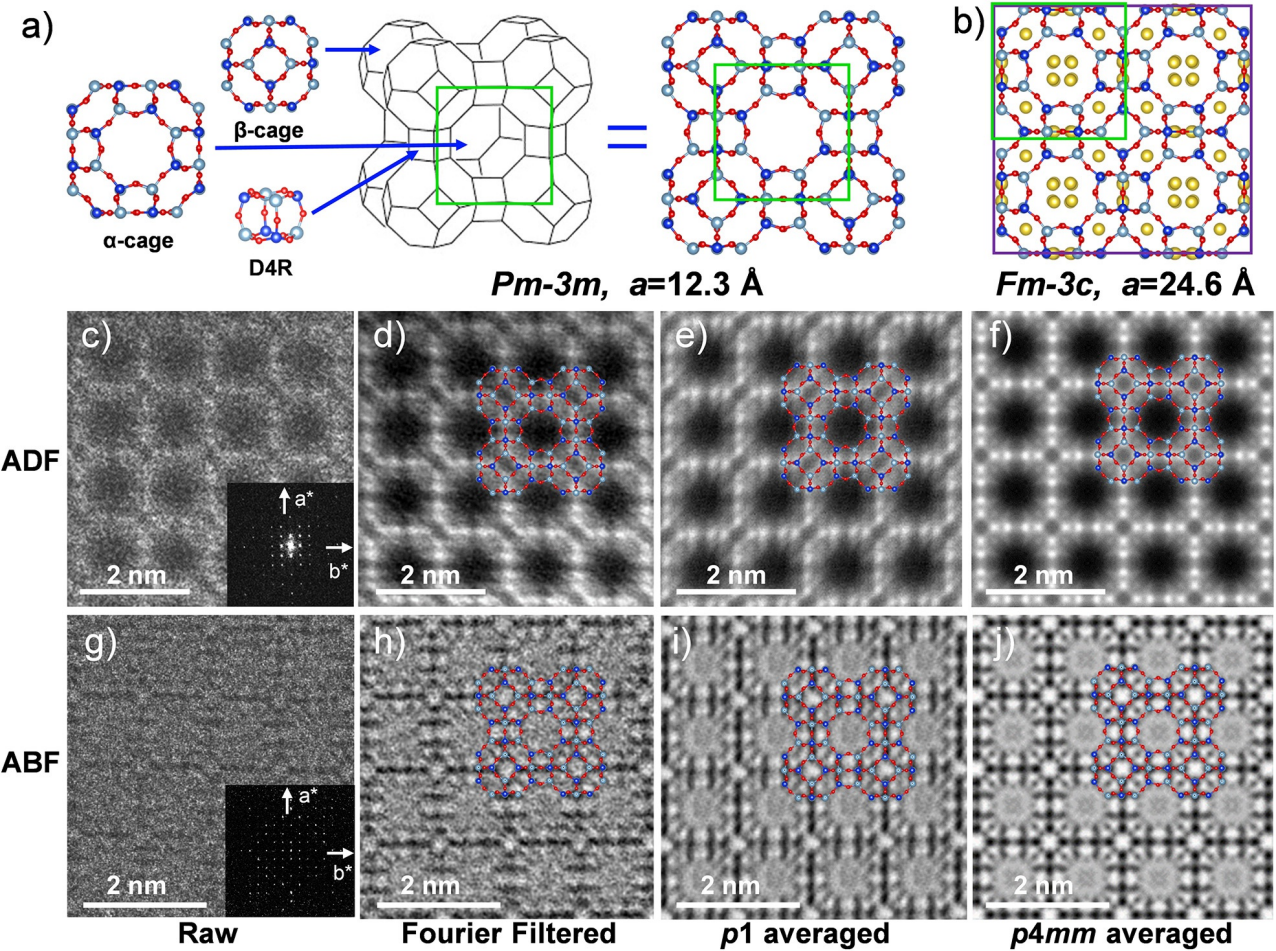
Framework structure of LTA, and ADF, and ABF images. a) Representation of the framework structure, with three SBU components of the α‐*cage*, β‐cage, and D4R, and the LTA unit cell (green square) of *Pm* $\bar{3}$ *m* (*a*=12.3 Å). b) *Fm* $\bar{3}$ *c* (*a*=24.6 Å), ADF and ABF images, c), g) raw images, d),h) Fourier filtered images, e),i) *p*1 symmetry‐averaged images, f),j) *p*4*mm* symmetry‐averaged images.

The best projection for S/TEM observation of the entire framework is ⟨100⟩, since many T‐O‐T bonds as well as S8Rs are perpendicular to the incident e‐beam, although Si and Al overlap in almost all projections along the principal zone axes. This direction of incidence is also the best to distinguish the three secondary building units (SBUs), α‐, β‐cages and D4Rs, in the structure. The model (with O‐, Al‐, and Si‐atoms shown by spheres in red, light blue, and dark blue, respectively), is shown in Figure 1 a. The structure can be explained as either *Pm* $\bar{3}$ *m* (*a*≈12.3 Å) without taking into account the Si and Al distribution or *Fm* $\bar{3}$ *c* (*a*≈24.6 Å), if two elements alternate, Na(1) and Na(2) positions reported by Pluth and Smith are marked on the *Fm* $\bar{3}$ *c* model (Figure 1 b), while Na(3) was omitted for clarity owing to its low occupation probability.

To acquire experimental data, crystals were tilted onto the required zone axis in TEM mode to minimize e‐beam damage. There have been some approaches for obtaining atomic‐resolution data of bare **LTA** and ion‐exchanged **LTA** by *C_s_*‐corrected STEM^14^ and *C_s_*‐corrected TEM.^15^ Among them, Yoshida et al. have observed the T atoms (T=Si and Al) and, to some extent, located extra‐framework cations (Cs and Na) in ZK‐4, which has the same **LTA** framework type but Si/Al=1.67. However, imaging both light cations (Na^+^)15b, 16 and O atoms remains so far inaccessible. In the present study, STEM mode was used for imaging, as it is advantageous over conventional TEM mode in terms of direct image interpretation, minimizing the presence of artefacts,^17^ and the possibility of acquiring different types of images in only one scan.

Figure 1 c–j presents a set of ADF and ABF images, starting from the observed raw data to the systematic processed images, specifically Fourier‐filtered, projection (plane group: PG) symmetry of *p*1 averaged, and *p*4*mm* averaged. Both averaged images were obtained from an area of 7 nm, which is proved sufficiently large to increase the SNR to retrieve images with improved spatial resolution. Figure 1 c–f shows the ADF images while Figure 1 g–j shows the ABF images. From the Fourier diffractograms (FDs) in the inset of the raw images (Figure 1 b and g), ABF gives a higher transfer information limit (1.26 Å) and finer details than ADF (1.56 Å). The signal to noise ratio (SNR) which is low in the raw data, is greatly enhanced by Fourier filtering as shown in Figure 1 d,h, which already show all T atoms of the framework. Furthermore, plane group symmetry averaging significantly improves the detectability, especially for the ABF image even by *p*1 symmetry average, which is just a translational average of the unit cell along [100] and [010]. It allows the observation of all T atoms, sodium in the 6SRs of the β‐cages and even oxygen bridges of the S4Rs; additionally, a faint contrast at the central part of the S8Rs can be also identified (Figure 1 i). When plane group symmetry *p*4*mm* (supporting information Figure S1 to see how this plane group was derived) is imposed, 4 sites which would be in agreement with the existence of Na^+^ in the S8Rs are revealed (Figure 1 j). Although the existence of image artefacts cannot be ruled out, the data obtained by this observation would be comparable to the report by Pluth and Smith (Figure 1 b). The validity of this symmetry will be discussed later.

**Figure 2:**
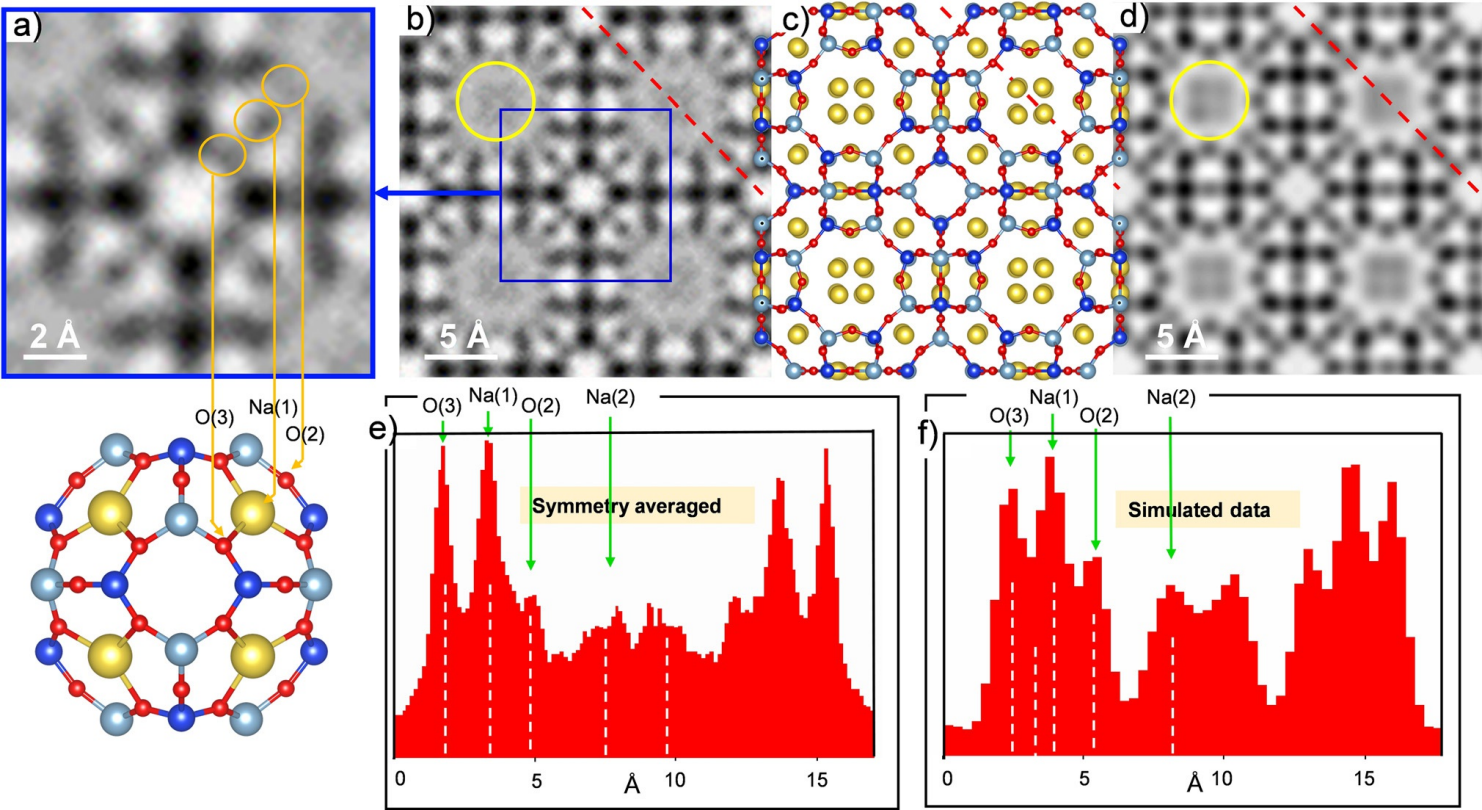
*C*_s_‐corrected STEM ABF images, corresponding framework structures, and intensity profiles. a) Enlarged ABF image of the β‐cage structure, with O and Na marked with orange circles. b) Experimental *p*4*mm* symmetry averaged ABF image of an LTA unit cell, with the same β‐cage as marked by a blue square, and Na cations marked by a yellow circle. c) Representation of the LTA model. d) Simulated ABF image. e) Intensity profile along the red dashed line in (b). f) Intensity profile along the red dashed line (d). Notations for atomic sites follow Pluth and Smith.

An enlarged ABF observation of the β‐cage, extracted from the *p*4*mm* symmetry averaged image (Figure 1 j), is shown in Figure 2 a together with a schematic model. The excellent match between the experimental data and the model is highlighted by orange circles and arrows, visualizing for the first time directly the O bridges of the S6Rs and S4Rs together with the Na^+^ cation. A symmetry‐averaged unit cell (*Fm* $\bar{3}$ *c*) is shown in Figure 2 b, with the projected atomic arrangement of the unit cell reported by Pluth and Smith (Figure 2 c) and the simulated data (for a thickness of 20 nm under the same experimental conditions used in the measurements, Figure 2 d). The intensity profiles from the experimental and simulated ABF images determined over the red dashed lines are shown in Figure 2 e,f. The intensity profiles clearly reveal the signal owing to the O bridges forming the S4Rs and S6Rs as well as the Na^+^ cations in the S6Rs. Furthermore, a weaker signal is observed in the S8Rs that is also in agreement with the model proposed by Pluth and Smith, who reported the existence of Na^+^ cations at four equivalent positions with approximately 1/4 occupancy. Such a low amount of Na^+^ is in agreement with the lower intensity of the signal from these cations, which are highlighted in Figures 2 b and d by yellow circles. An aspect that cannot be completely ruled out is that at such a low *q* value artefacts may exist, that could influence this signal. Thus, the similarities between the experimental and simulated data suggest the existence of Na^+^ at those S8Rs, but further conclusions cannot be extracted due to the low Na^+^ occupancy.

To confirm the validity of this image analysis, a closer comparison was made between experimental raw images in ADF/ABF modes and the simulated images under the same conditions of electron‐dose per unit area, 3000 e^−^ Å^−2^ (Supporting Information, Figures S2–S4). The results obtained confirm that even images taken at such low SNR contain a significant amount of information in terms of spatial resolution and atomic sensitivity.

In the present work, Na‐**LTA** was used as a proof of concept, but in general terms the direct observation of O‐atoms in the framework is of paramount importance in catalysis, not only for distinguishing distances between Si−O and Al−O in the tetrahedra, but also for giving information on the interaction between the framework O atoms and cations responsible for catalytic performance. Since this can be achieved with very high local resolution (even under symmetry averaging conditions), the information obtained extends substantially beyond what can be obtained by XRD or 3D‐EDT. Additional information was also recorded along another main crystallographic axis. The STEM‐ADF and ABF images recorded along the [110] incidence are shown in the Supporting Information, Figure S5. Although O bridges are not seen in this case, the presence of Na(1) in the S6Rs is clearly observed.

**Figure 3:**
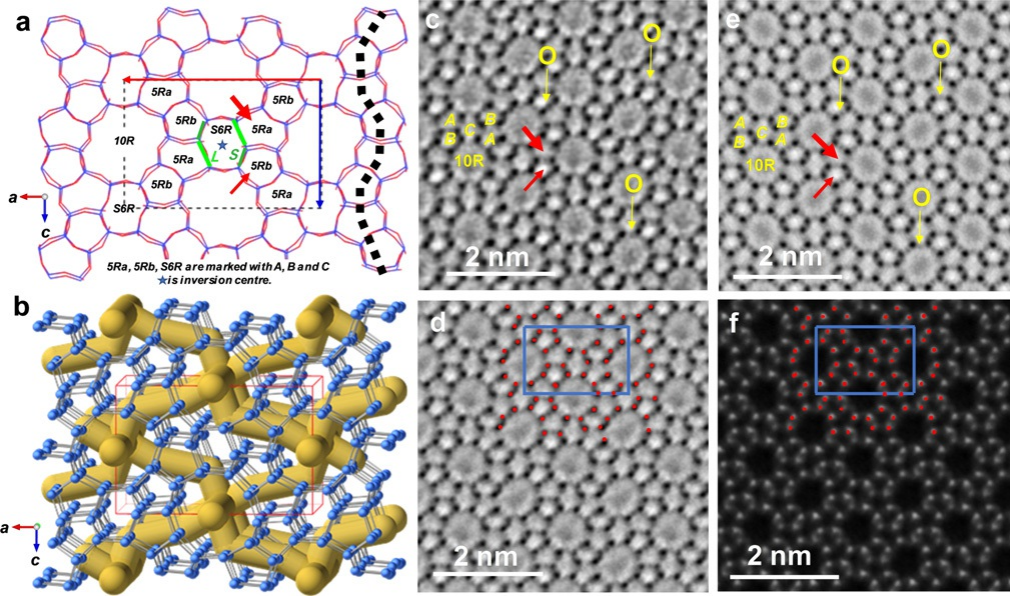
a) Drawing of the MFI projected structure along [010]. Arrows point to the large, 5Ra and small, 5Rb membered rings. L and S correspond to the long and short distances between the T atoms. The star denotes a center of inversion. b) 3D structure with a straight channel along [010] and sinusoidal channels in the a‐c plane. c) *C_s_*‐corrected STEM‐ ABSF (average background subtraction filtered image) taken with the [010] incidence. d) *p*1 symmetry averaged image, e) *p2gg* symmetry‐averaged image, f) *p*1 symmetry‐averaged *C_s_*‐corrected STEM‐ADF image.

**Figure 4:**
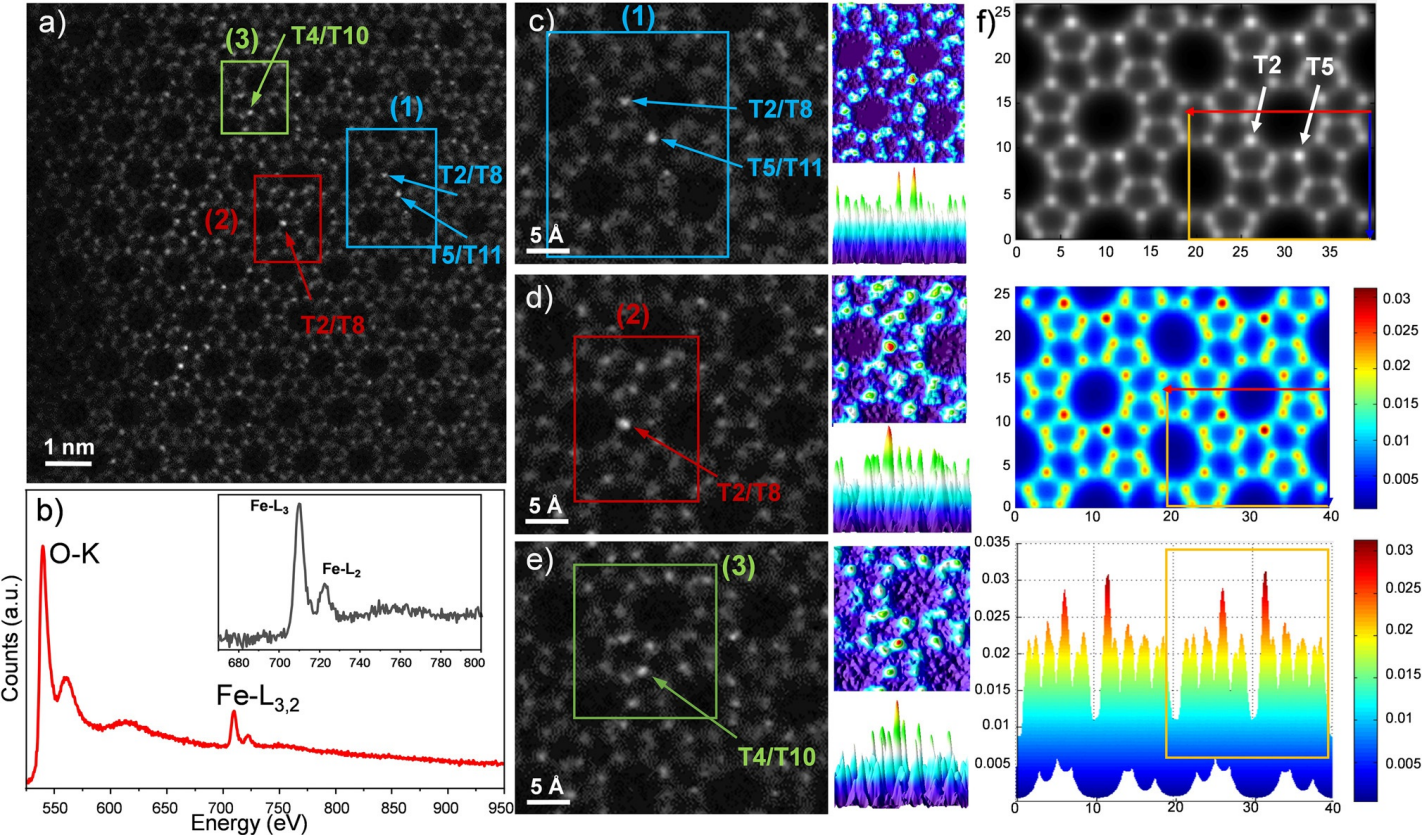
*C_s_*‐corrected STEM ADF images and EEL spectrum of Fe‐MFI. a) High‐resolution ADF image. b) EEL spectrum. c)–e) Enlarged images corresponding to the three regions marked by rectangles in (a) together with surface plots of 2d‐intensity distribution map, where bright dots in (a), (c), (d), and (e) are marked by arrows with T‐site symbols. f) Simulated images of Fe‐MFI, where two single Fe atoms are located at T2 and T5 sites corresponding to two Fe atoms per unit cell, under the conditions of probe size: 1.0 Å and specimen thickness: 105 Å.

The structure of ZSM‐5 (**MFI** structure type) was solved by Kokotailo et al.^18^ as an orthorhombic structure with space group *Pnma* and lattice parameters *a*=20.09 Å, *b*=19.73 Å, and *c*=13.14 Å. Projection along the *b*‐axis allows the best visualization of the framework exhibiting: 1) two types 5‐membered rings (5Rs) of different size; a large 5Ra and a small 5Rb, which are alternately arranged along the c‐axis; 2) single 6‐membered rings (S6Rs), and 3) the largest pores formed by 10‐membered rings (10Rs) (Figure 3 a,b; Supporting Information, Figure S6). **MFI** contains a three‐channel system: one straight channel along the b‐axis, and sinusoidal channels in the *ac*‐plane (Figure 3 b), marked by a dashed black line in Figure 3 a. The S6Rs have long and short edges marked by bright and dark green lines with L and S characters and the center of inversion is marked by a star. The projection along [010] is the most appropriate direction to observe all the framework atoms, although two T‐atoms in different sites are almost overlapped, as shown in Figure 4.

Before *C_s_*‐corrected STEM became available, individual T‐atomic columns could not be observed separately, therefore 5Ra and 5Rb could be recognized only as large and small pores, together with a center of inversion at the center of the S6Rs,^19^ making further analysis impossible for single atom replacement in the framework.

With the aim of observing the entire framework, including bridging O‐atoms, ABF conditions were chosen since the signal from lighter elements can be enhanced in this mode compared to the traditional BF,6c as already presented for **LTA**. Figure 3 c shows the average background subtraction filtered (ABSF) image of a Fe‐**MFI** crystal. Despite the low SNR, it is possible to identify the four types of rings, 5Ra, 5Rb, S6R, and 10R. Furthermore, many O bridges are also observed, indicated by yellow arrows (with no further data treatment except for ABSF). Nevertheless, in order to further enhance the SNR, the image was symmetry averaged by *p*1 (Figure 3 d) and *p*2*gg* (Figure 3 e), which is the projected symmetry along the [010] orientation for *Pnma*. As expected, and especially for *p*2*gg*, all T atomic columns are located together with sharper definition of the oxygen positions. For clarity, O bridges are only marked in Figure 3 c and e and T atoms are marked by red spheres in Figure 3 d, while the unit cell is indicated by a blue rectangle.

Among the many advantages of working in STEM mode, it is possible to simultaneously record information on light elements with an ABF detector and elements with different atomic number (Z‐contrast) with an ADF detector, using only one scan. All T‐sites are clearly observed (marked by red dots) in the *p*1 averaged ABF image (Figure 3 d), confirming that all T‐sites and their geometrical arrangement exactly fit those shown in Figure 3 a. If Fe atoms would replace Si atoms at certain preferential crystallographic positions, it would then be possible to distinguish bright dots for these sites in the *p*1 averaged ADF image (Figure 3 f). Although it is possible to recognize slight intensity fluctuations among the T‐sites in Figure 3 f, it is still necessary to have solid experimental evidence in order to corroborate the presence of Fe atoms and their location without any symmetry averaging.

For this reason, *C_s_*‐corrected STEM‐ADF analysis was carried out over the raw data for very thin regions of Fe‐**MFI** crystals (Figure 4). In this case, there is evidence of bright spots in the raw ADF image (Figure 4 a). Considering that this image is Z‐dependent, Fe is expected to produce a stronger signal in comparison with Si, thus Fe atoms would be detected as brighter dots at different T sites. To corroborate the presence of Fe that isomorphously substituted the Si (as no particles, iron aggregates/precipitates, were observed), electron energy loss spectroscopy (EELS) analysis was carried out on every crystal analyzed, with two clear distinct signals from the O‐K edge observed from the zeolitic framework and the Fe‐L_3,2_ edge (Figure 4 b). Furthermore, infrared (IR) spectra for Si‐**MFI** and Fe‐**MFI** exhibit significant differences (Supporting Information, Figure S7), with a signal at 3725 cm^−1^ corresponding to Si−OH bonds present for Si‐**MFI**, which appears almost at the same value for Fe‐**MFI**, 3723 cm^−1^. Furthermore, for Fe‐**MFI**, another strong band appears at 3617 cm^−1^, assigned to bridging OH groups Si−OH−Fe.

Following the T‐site notation adopted by the Structure Commission of the International Zeolite Association, three regions are marked with rectangles in Figure 4 a: 1) for T2/T8 and T5/T11, 2) for T2/T8, and 3) for T4/T10, where the brightest spots (atomic columns) are indicated by arrows. Intensity analysis was carried out over these three regions (Figures 4 c,d,e) using ImageJ software^20^ to create the intensity surface plot, in which the stronger signals are more clearly identified with respect to the framework. For this analysis, very small regions were chosen to minimize the possible effect of thickness variations. For the three cases, the experimental ADF data are presented, with arrows marking specific T sites that have a more intense signal, suggesting that Fe may preferentially occupy these sites. In each case, a thermally colored 3D surface plot is also shown, extracted from the rectangles (top plot), together with the perpendicular observation (bottom plot), it is clear that the maxima observed correspond to the brightest spots. In every case, there is unambiguous evidence of a more intense atomic column that, assuming the thickness can be taken as constant in such a small area, may be interpreted as the partial substitution of Si by Fe along those columns. To corroborate this observation, image simulations were carried out by building a supercell of 39.886×26.048×198.710 Å^3^, with one Fe atom per column/unit cell introduced at the T2 and T5 sites. Different collection angles were tested, but to match the experimental conditions, an inner angle of 30 mrad was chosen. Figure 4 f top corresponds to a thickness of 105 Å and a probe size of 1 Å, the effect of the crystal thickness on the simulated images is presented in the Supporting Information, Figure S8, observing no detectable variations in the intensity plots. The two brightest sites, where the Fe atoms were placed, are marked by two arrows in the top image. The surface plot, based on thermal coloring is presented in Figure 4 f middle, and the perpendicular observation (similar to the experimental data) appears in Figure 4 f bottom. For reference, a rectangle that corresponds to a unit cell is marked in the three images. Direct comparison between the experimental and simulated surface plots proves the presence of at least one Fe atom replacing a silicon per unit cell, with the zeolitic framework remaining intact. The possible influence of the location of Fe atoms at different depths along the same column was also evaluated observing no significant variations on the intensity profiles (Supporting Information, Figure S8c). An additional simulation without the presence of Fe is displayed in the Supporting Information, Figure S8c exhibiting a much more homogeneous intensity in contrast to those where Fe is present.

After analysis of several images, it is not possible to identify any preferential sites for Fe incorporation, as different T sites were found to be occupied by Fe. On the other hand, if preferential siting would exist (as may be achieved by different synthesis protocols), the data show that it would be possible to directly detect this and then correlate such preferential siting with specific catalytic behavior. The data suggest that silicon substitution by iron atoms occurs rather statistically for this specific example. This will be further discussed in the Supplementary information that includes de DFT calculations (Supporting Information, Figures S9–S14 and Tables S1–S3).

### Structural Study of Na‐LTA and Fe‐MFI by Electron Diffraction

Three‐dimensional electron diffraction tomography (3D‐EDT) data, selected area electron diffraction (SAED) and precession electron diffraction (PED) patterns were recorded for nano‐crystals (obtained by deeply crushing the parent material) of Na‐**LTA** in the volume range of (100–400 nm)^3^.

By analyzing such a small volume, it was intended to observe any possible deviations from the theoretical *Fm* $\bar{3}$ *c* symmetry that could be hidden by a general averaging of larger crystallites. This concern was raised due to impossibility of placing all Na^+^ with occupation probability=1 in the framework and still keeping cubic symmetry (see the Supporting Information, Figure S15 for the 3D‐EDT data).

Based on cubic symmetry with *a*≈25.1 Å (as we have observed three‐fold symmetry in ED patterns taken from the large volume mentioned above along two independent incidences corresponding to cubic ⟨111⟩) the following reflection conditions were obtained: *hkl*: *h*+*k*=2*n*, *k*+*l*=2*n*, *h*+*l*=2*n*; *0kl*: *k*,*l*=2*n*; *hhl*: *h*,*l*=2*n h00*: *h*=2*n*, where *h*, *k*, and *l* are permutable, and the possible extinction symbol would be *F*_ _*c*. The corresponding possible space groups were *F* $\bar{4}$ 3*c* (No. 219) and *Fm* $\bar{3}$ *c* (No. 226). A few weak extra reflections at odds with *Fm* $\bar{3}$ *c*, such as $\bar{1}$ 1$\bar{1}$ and 131, were observed in the 3D‐EDT and in the SAED patterns in different crystals (Supporting Information, Figures S15–S17). However, both disappeared by tilting along the −*hh*−*h* axis and they were not observed using PED (Supporting Information, Figures S18). Therefore, both reflections can be explained by multiple scattering. Other possibilities than cubic, including a mixture of very small oriented domains with different variants of tetragonal or orthorhombic symmetries, need to be carefully checked in future. In the DFT calculations, tetragonal cases with *a*=*b*=*c*≈25.1 Å were discussed.

**Figure 5:**
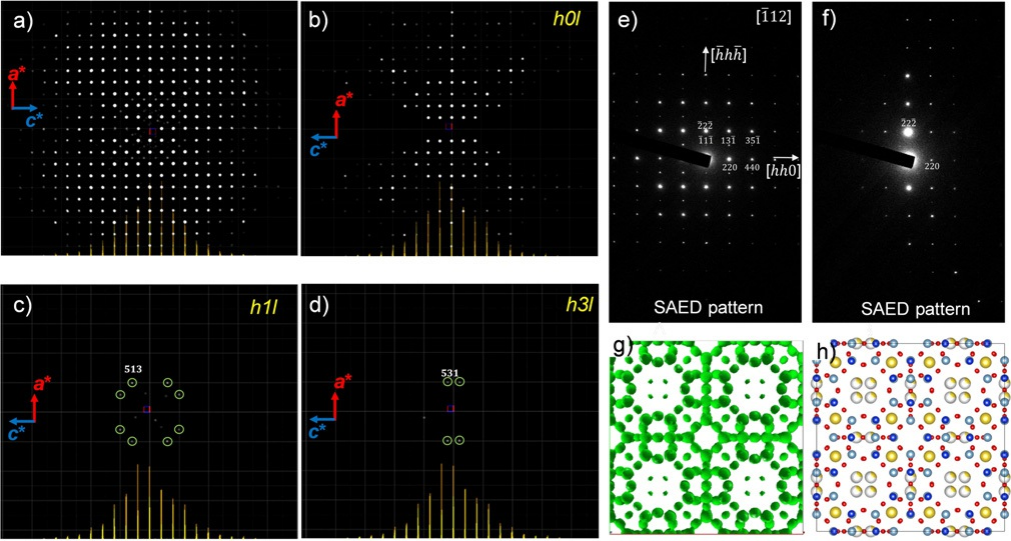
3D‐EDT data, SAED patterns, and structure solution of Na‐LTA. a) Projected diffraction pattern along [010], b*. b)–d) Three slices containing *h0l*, *h1l*, and *h3l* reciprocal planes. e),f) Selected‐area electron diffraction (SAED) patterns with [$\bar{1}$ 12] incidence and slightly tilted along −*h*, *h*,−*h*. g),h) Electrostatic potential map and crystal structure with OPs.

As there have been some reports of zeolites with the same **LTA** framework‐type with Si/Al>1,^21^ symmetry deviations from *Fm* $\bar{3}$ *c* were also studied through 3D‐EDT by using as small a crystal as possible down to 40 (unit cell)^3^, which is currently the minimum size to obtain structure solution from Na‐**LTA**, because of weak intensities for *hkl* reflections with high *q* (*q*=4π sin*θ*/*λ*). However, no apparent deviations were observed (Figures 5; Supporting Information, Figure S15). From the ED approach, a possibility of small oriented domains with tetragonal *P*4/*ncc* (no three‐fold axes) and *a*=*b*=*c*≈25.1 Å cannot be completely eliminated; although, at the current state, due to a limitation in the precision in determining the positions of the diffraction spots in such a small volume do not allow further conclusions. If space group *P*4/*ncc* with *a*≈25.1 Å was assumed, all Na cations can be explained without introducing occupation probabilities; however, the Si/Al ratio will be 3/1 to avoid Al−O−Al bonding.

The structure of Fe‐**MFI** was determined through 3D‐EDT, SG *Pnma* with lattice parameters of *a*=20.6 Å, *b*=20.6 Å, *c*=13.9 Å, *α*=90.3°, *β*=90.7°, *γ*=90.3° from the observed reflections and the extinction rules. The structure was solved and all framework atoms were identified. From the electrostatic potential map, no preferential occupancies of Fe on different T‐sites were observed (Supporting Information, Figure S19) from the high‐quality ED data obtained from rather large volume, in agreement with the STEM analyses.

## Conclusion

Through *C_s_*‐corrected STEM coupled with ADF and ABF image modes, oxygen atoms and Na^+^ cations are directly observed, together with all framework T‐atoms, in zeolite Na‐**LTA**. This atomic resolution is highly remarkable, since Na‐**LTA** has Si/Al ratio of about 1.0 and is one of the lowest framework density zeolites, making it one of the most e‐beam‐sensitive materials. Direct oxygen visualization represents a substantial increase in the information limit in terms of sensitivity as well as spatial resolution. It also points the way to the analysis of the local structure around heteroatoms in a zeolite framework, including the distortion of the oxygen tetrahedron. In Fe‐**MFI**, oxygen bridges are also clearly located, proving the feasibility of the ABF method as an improved technique for the analysis of e‐beam sensitive materials.

An ongoing discussion on whether and under what circumstances Fe is indeed located in zeolite frameworks or present as extra‐framework species can be concluded. Under the right synthesis conditions, iron can be incorporated into the framework, and in future work it will be able to clearly follow the pathways for the formation of extra‐framework species. This has highest relevance for important catalytic reactions, as it has been claimed the framework and extra‐framework species have rather different catalytic properties. Our data provide clear evidence of Fe on tetrahedral sites within the zeolite structure which are clearly distinguished as single atoms by the ADF technique. This extends substantially the limits of resolution and sensitivity that can be achieved with electron microscopy techniques for this important class of materials.

These results represent crucial progress in zeolite science for the analysis of structural defects down to single heteroatoms or point defects, which are responsible for the creation of catalytic sites and/or enhanced thermal stability. The present study provides the blueprint for how to achieve this level of information for other members of the technically very relevant classes of zeolites. With such a high level of atomic visualization, new structures which have been computationally modelled and that contains different kinds of defects or intergrowths will be able to be elucidated.

## Conflict of interest

The authors declare no conflict of interest.

## Supporting information

As a service to our authors and readers, this journal provides supporting information supplied by the authors. Such materials are peer reviewed and may be re‐organized for online delivery, but are not copy‐edited or typeset. Technical support issues arising from supporting information (other than missing files) should be addressed to the authors.

Supplementary
