# Supplementary material for: Direct Atomic‐Level Imaging of Zeolites: Oxygen, Sodium in Na‐LTA and Iron in Fe‐MFI
Source: Angew Chem Int Ed Engl. 2020 Jul 20;59(44):19510–7. doi: 10.1002/anie.202006122 (PMC7689718; doi:10.1002/anie.202006122)
Supplement: Supplementary file 1 — Supplementary [file ANIE-59-19510-s001.pdf]

Angewandte Chemie
Eine Zeitschrift der Gesellschaft Deutscher Chemiker

Supporting Information

Direct Atomic-Level Imaging of Zeolites: Oxygen, Sodium in Na-LTA and Iron in Fe-MFI

Alvaro Mayoral, Qing Zhang, Yi Zhou, Pengyu Chen, Yanhang Ma, Taro Monji, Pit Losch, Wolfgang Schmidt, Ferdi Schüth, Hajime Hirao, Jihong Yu, and Osamu Terasaki**

anie_202006122_sm_miscellaneous_information.pdf

Supporting Information

Experimental section

Sample preparation:

Na-LTA: Based on Charnell's report, synthesis conditions were optimized for synthesis of Na-**LTA** single crystals. $NaSiO_3/9H_2O$ (100 g) was dissolved into a mixture of distilled water 700 ml and triethylamine (TEA) 100ml at 50 °C. $NaAlO_2$ was independently dissolved into the same amount of the mixture of TEA and water at 5 °C. After filtering both solutions, using a PTFE membrane filter with 0.2 µm pores and aging for 2 weeks, $NaAlO_2$ solution was added dropwise to the $NaSiO_3/9H_2O$ solution under stirring to finally obtain a uniform gel. Afterwards, it was possible to observe a clear a two-phase solution formed by an upper semitransparent liquid and a denser gel at the bottom; the gel volume decreased with synthesis time. Crystallization finished in 1~3 weeks depending on the temperature. The best temperature for single-phase crystallization was 85 °C. The final product obtained consisted of single crystals of 50 to 60 µm.

Fe-MFI: High crystalline Fe-**MFI** (Silicalite-1) was synthesized in Teflon lined stainless steel autoclaves. 20 mL of water was added to 0.333 g of TPABr and stirred until complete dissolution. Then, 0.666 g of NH_4F and 0.334 g of $Fe(NO_3)_3 \times 9\ H_2O$ were added to the same solution. Finally, 3.69 mL of TEOS was slowly (within 2 minutes) added to the solution. The almost milky suspension obtained was then stirred (700 rpm) for 2 h at room temperature. After this aging treatment, the synthesis gel was transferred into a Teflon lined stainless steel autoclave. Hydrothermal synthesis was performed at 185 °C for 7 days. Afterwards, a slightly colored material was obtained after filtration, which was deeply washed, 3 × 30 min with 6 N HCl at room temperature to remove any possible FeO_x contaminants. Calcination was carried out at 450 °C for 15 h. The calcination led to the final Fe-**MFI** which possesses some acidic protons as we verified by FTIR (DRIFTS).

The samples were synthesized in fluoride medium to avoid to the maximum any such Si-dislocations. To avoid the presence of any extra-framework Fe-species several acidic washing steps were undertaken to remove them at best.

Electron microscopy observation

STEM data were collected at 300 kV with the recently installed JEM-ARM300F microscope equipped with cold FEG and double C_s correctors at the Centre for High-resolution Electron Microscopy (*ChEM*), ShanghaiTech University. Prior any observations the STEM corrector for the electron probe was corrected using amorphous carbon, assuring a spatial point resolution of 0.7 Å.

The samples were prepared by deeply crushing a small amount using mortar and pestle for several minutes. The powder was then dispersed in ethanol to create a suspension with the finely divided crystallites. Few drops of the suspension were placed onto holey carbon copper grids.

Before analyses, the beam current was carefully measured using a faraday cup installed at the projection screen.

For both Na-**LTA** and Fe-**MFI**, ABF conditions were satisfied by introducing a dedicated ABF aperture and by using the BF detector as a data collector. For ADF in both cases the inner collection angle was 30 mrad and the hybrid ADF detector from JEOL was used.

For all data 1024 × 1024 pixel images were recorded and exposure time was varied from 3µs up to 12 µs not exceeding an electron dose of ≈ 3000 $e^-/Å^2$ for **LTA** and ≈ 4000 $e^-/Å^2$.

For every case the convergence semiangle selected was 17 mrad.

EM simulations were performed using QSTEM software[1] based on the multislice method. For the **LTA** a supercell was built with the parameters 24.55 × 24.55 × 294.66 $Å^3$ while for the **MFI** structure the

unit cell was 39.886 x 26.048 x 198.710 $Å^3$. Experimental data was treated by using Digital Micrograph software and CRISP software[2]. Selected area electron diffraction (SAED) and 3D ED tomography (3D-EDT) of Na-**LTA** and Fe-**MFI** were conducted using JEM-2100Plus in TEM mode operated at 200kV. The angle range varied between -60.4° to 62.2° using a step size 0.2°. Data collection was acquired through EDT-Collect and processed using EDT-Process software.

FTIR (DRIFTS).

Infrared spectra were collected in DRIFTS mode using 2–5 mg sample and a modified Harrick DRIFTS cell with KBr windows. Spectra were measured in the 4000–1000 cm^{-1} range with a 4 cm^{-1} resolution and averaging 250 scans using a Nicolet Magna-IR 560 spectrometer equipped with a MCT detector (Thermo Nicolet, Waltham, MA, USA). Samples were dried at 523 K under flowing dry nitrogen (10 mL min^{-1}) for 30 min. Thereafter, the collection of spectra started at 523 K. All spectra were normalized by the intensity of the Si–O–Si overtones (2100–1750 cm^{-1}). IR data were treated with Opus software and finally plotted using Origin software.

Figure S1

Deduction of the plane group symmetry

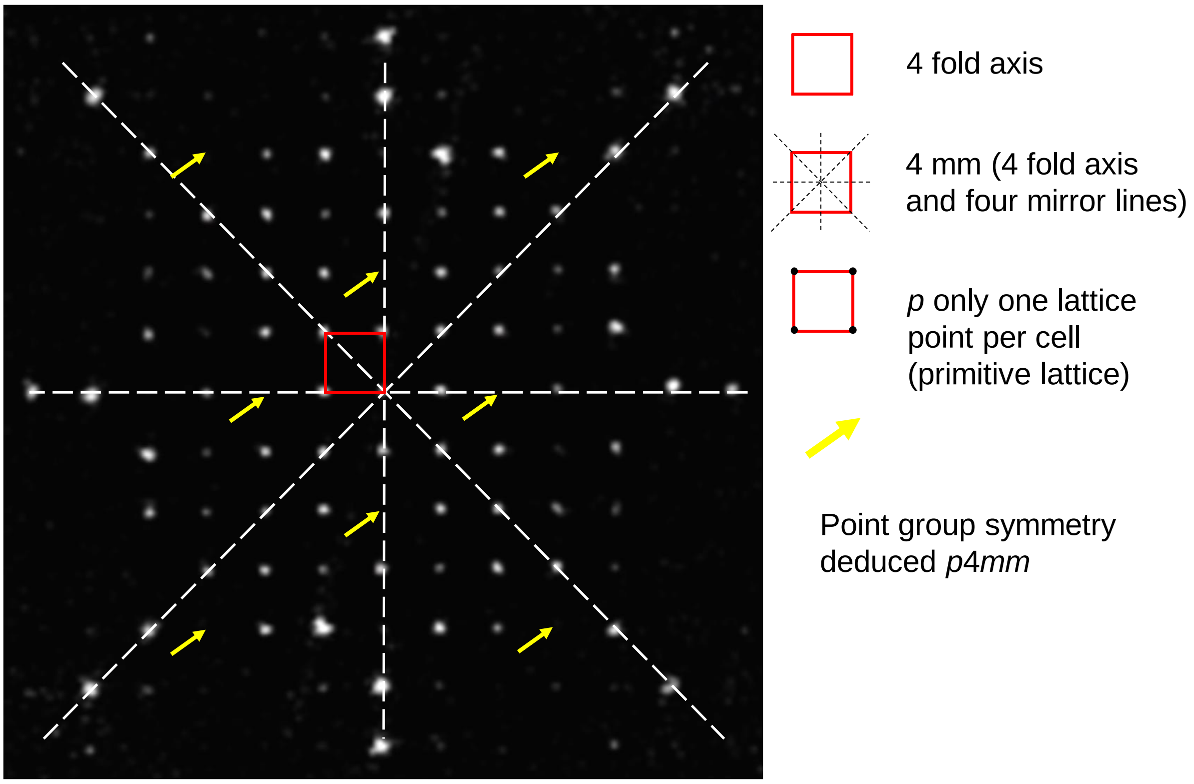

Figure S1. Fourier Diffractogram along the [001] orientation with the symmetry operations denoted. The yellow arrows indicate the schematic absences.

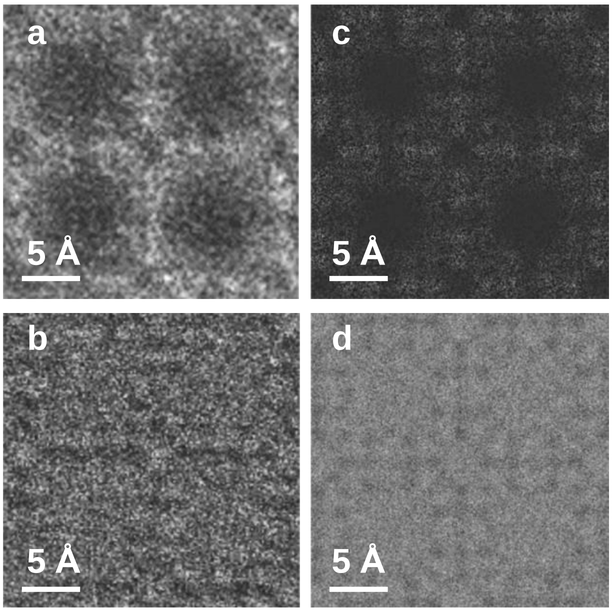

Figure S2. C_s-STEM ADF and ABF images of Na-LTA of [001] incidence. a) and b) Raw experimental ADF and ABF images, respectively. c) and d) Simulated ADF and ABF images that take into account similar electron dose of 3000 $e^-/Å^2$.

In general, simulated EM images tend to have much better quality (even for the same experimental conditions) than those obtained in the electron microscope. This is because factors such as scanning distortion, a small miss-tilt of the crystal, beam damage and the noise introduced by low doses are commonly neglected. Figure S4a and S4b show the experimental raw ADF and ABF images. In order to make a closer comparison between experimental and simulated data, the dose of electrons per area (3000 $e^-/Å^2$) was introduced into the simulated ADF and ABF images (Figure S4c and S4d). Although the SNR is very low, this analysis illustrates how much information can be extracted even from highly noisy data, indicating that it is possible to obtain atomic resolution information even at such low dose conditions. Further analysis that corroborates this result is presented in Figure S3, where the same image analyses carried out for the experimental micrographs was also applied to the simulated (noised) images. Noised images together with the Fourier filter and symmetry averaged images are shown for both detectors in Figure S3.

Image analysis and image simulation

EM image simulations were performed using QSTEM software.[1] Experimental data were treated by using Digital Micrograph software and CRISP software.[2]

For Na-LTA, a supercell of 24.55 Å × 24.55 Å × 294.66 Å was created. Simulation parameters were set the same as the experimental ones, keeping the spherical aberration below 1 micron. Figure S2 compares the annular dark field (ADF) experimental data for one unit cell (24.55 Å) (Figure S2a), with the simulated data (Figure S2b, for a given thickness of 20 nm). An experimental ABF image is presented in Figure S2c, while the simulated one appears in Figure S2d. The low amount of electrons used in the experimental and to simulate the data, gave as a result very noisy images that, however; still contain lots of information. Figure S3 show the ADF and ABF images of several unit cells obtained by introducing the same electron dose as the experimental conditions. After Fourier filtering, as it can be clearly appreciated, the amount of information was significantly enhanced. Symmetry averaging analysis provided a much higher degree of information which allowed the visualization of the T atoms and the Na at the 6Rs, and of all atoms, including O bridges and different Na cations in the ABF data. For image simulations the model constructed was based on the previous work by Pluth & Smith[3], thus Na cations at coordinates 0.0996, 0.0996, 0.0996 with an occupancy of 1 as well as Na cations with ¼ of occupancy at the 8Rs were incorporated.

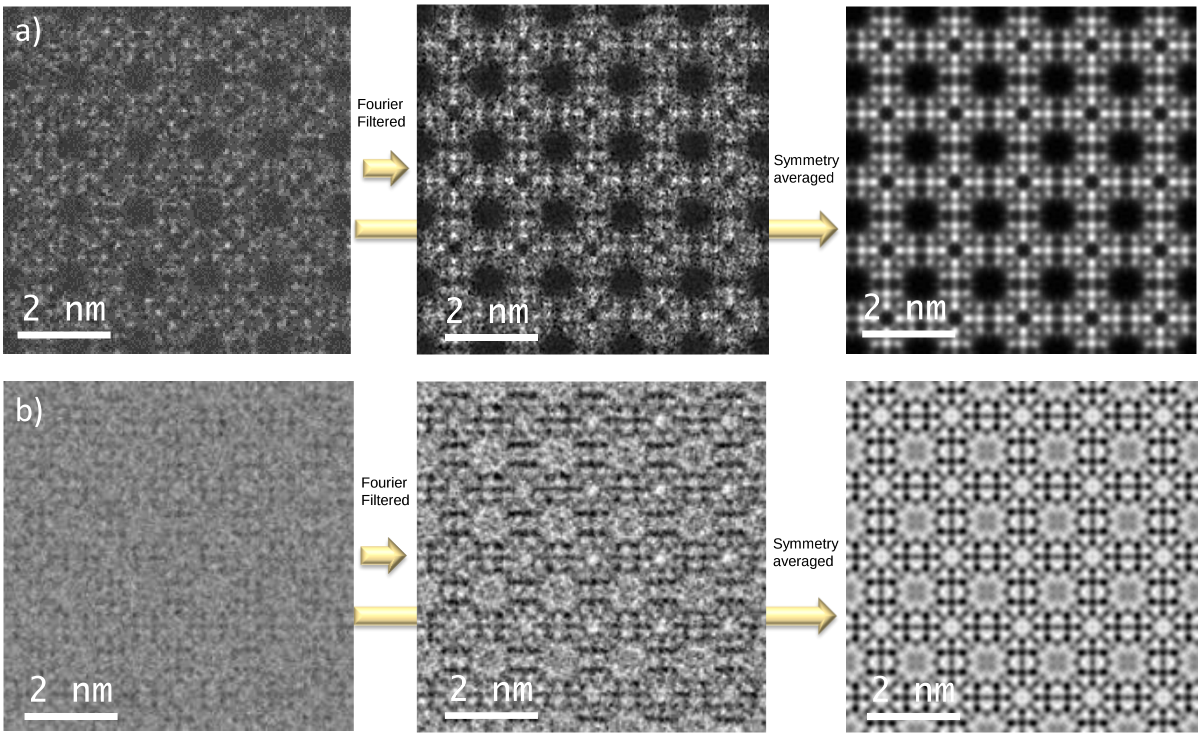

Figure S3. a) ADF image analysis: the simulated Na-LTA image and the Fourier filtered and symmetry averaged images. b) ABF image analysis: the Na-LTA image and the Fourier filtered and symmetry averaged images.

The differences in the treatments for increasing the visibility of the original ABF image are provided here. Each treatment for original image, two filter methods, Fourier- and Wiener-filtered images (**a** and **b**) and images obtained via, two projected symmetry averaging (plane-symmetry) for the image with *p*1 and *p*4*mm* (**c** and **d**) are shown here.

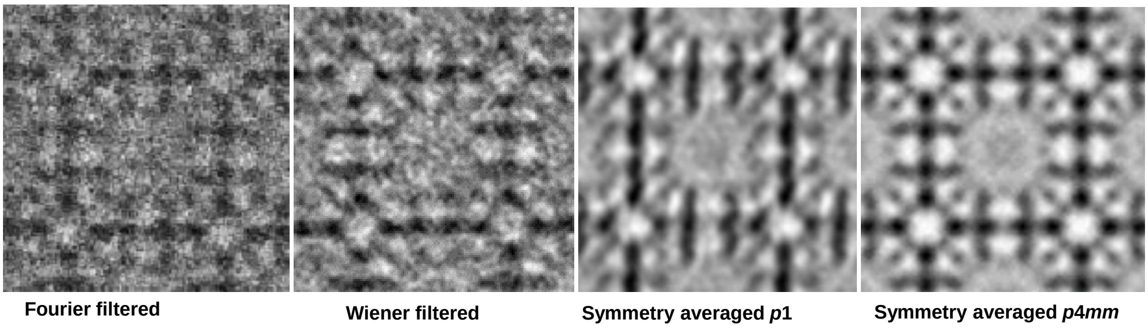

Figure S4. Different micrographs obtained via various image processing approaches.

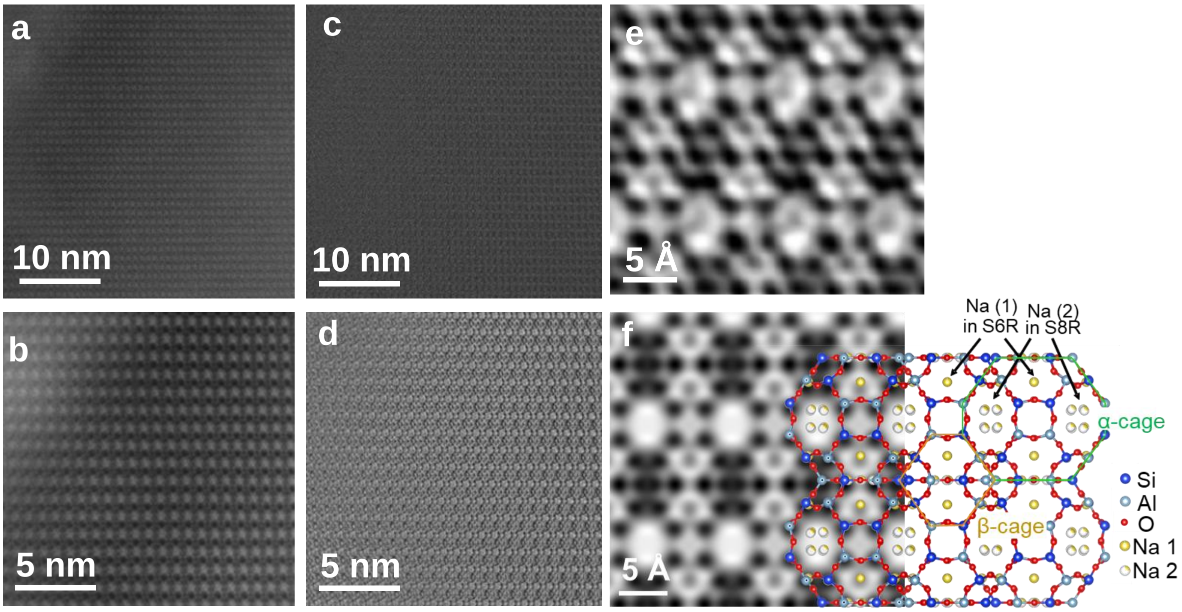

Figure S5. C_s-corrected STEM-ABF images of Na-LTA taken along the [110] zone axis. a) Raw ADF micrograph. b) magnified ABSF image of the ADF data. c) ABF micrograph. d) magnified ABSF image of the ABF data. e) enlarged observation of the porous arrangement from d. f) *p2mm* symmetry averaged ABF image with a schematic representation overlaid.

Basic structure of MFI

MFI zeolite has an orthorhombic structure with space group *Pnma* and lattice parameters; a = 20.09 Å, b = 19.73 Å and c = 13.14 Å. The projected framework structure along the b-axis has plane group symmetry of *p*2*gg* (Figure S6a) and contains two different 5-membered rings (5Rs) in size, large 5Ra and small 5Rb which are alternately arranged along c-axis; single 6-membered rings (S6Rs) and the largest pores with 10-membered rings (10Rs), Figure S6b. This projection is the best one in order to visualize the **MFI** framework; however, some different T atoms are on the overlapped along the same columns and therefore are not visible. By looking along the *c*-axis, it is possible to locate all T-sites which has been labeled consistently with the IZA database nomenclature (Figure S6c).

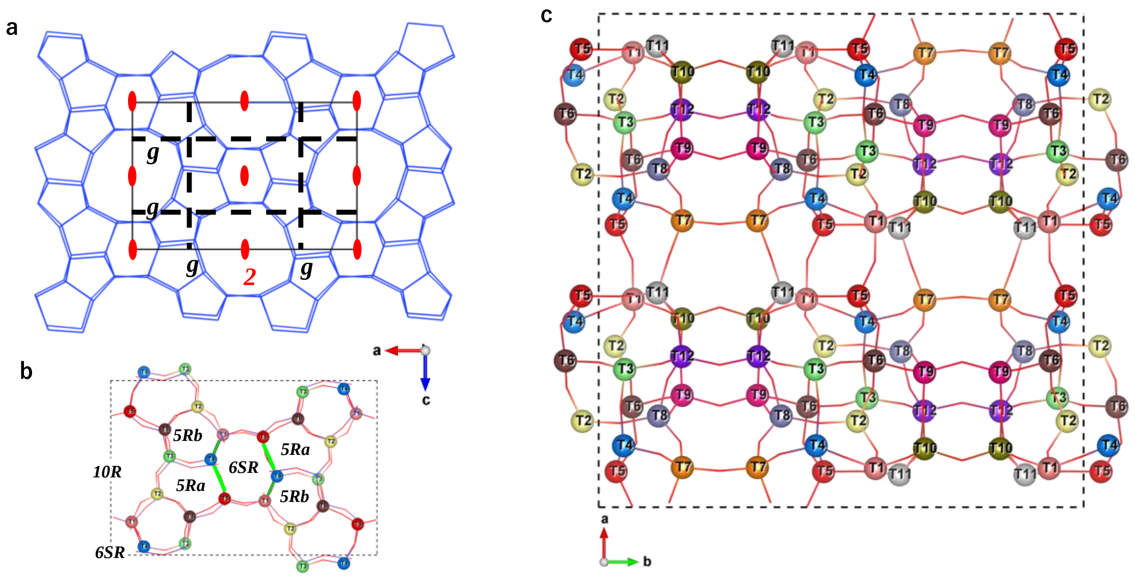

Figure S6. Basic structure of MFI and different T-sites. a) and b) projected framework structure along the *b*-axis with symmetry elements of plane group *p*2*gg*, and four different rings 10R, S6R, 5Ra and 5Rb. c) all different T-sites viewed along the *c*-axis.

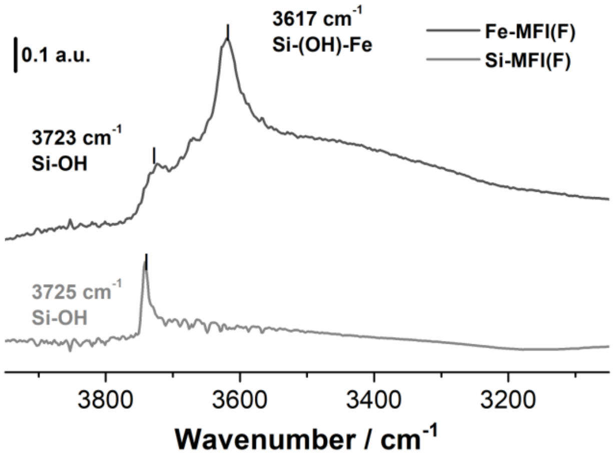

Figure S7. Infrared profiles of Fe-MFI and pure silica Si-MFI.

Fe-MFI simulations of different thickness

In order to corroborate the possiblity of identifying Fe that would be incorporated into the framework, only one Fe atom per unit cell was incorporated into the T2 and another into the T5 sites. See Figure S8a. Simulations for different thicknesses using an inner angle of of 30 mrad, a convergence ange of 17 mrad and probe size of 1Å are depicted in Figure S8b. For thicknesses over 135 Å no substantial diffferences were observed. Addtitional simulations, Figure S8c, presents the influence of the Fe location algong the axes of observation, observing no substantial changes in the intensity produced by the Fe atoms. For comparison, exprimental data on bare MFI zeolite, without Fe is also presented together with the correspondent iamge simulations.

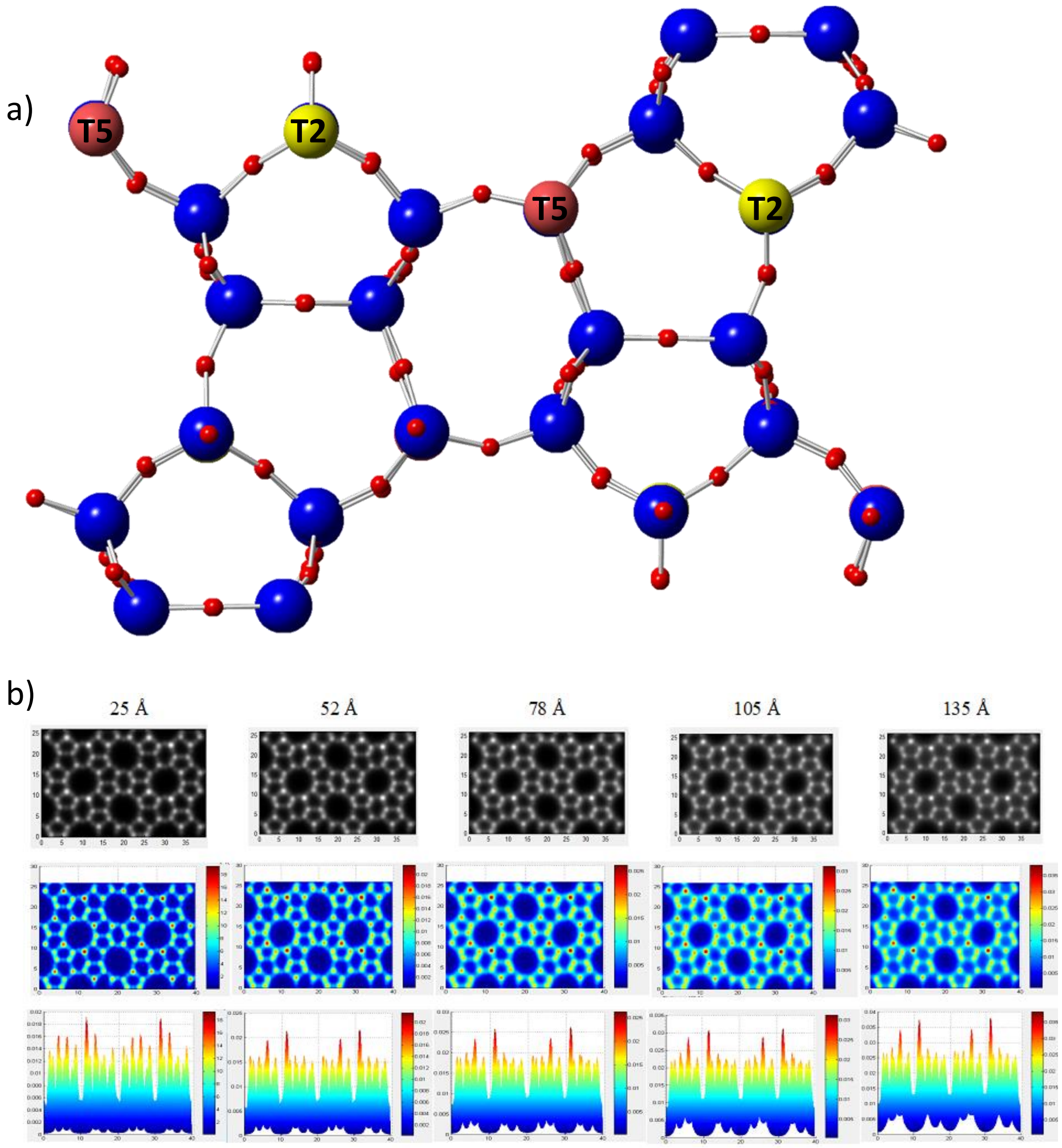

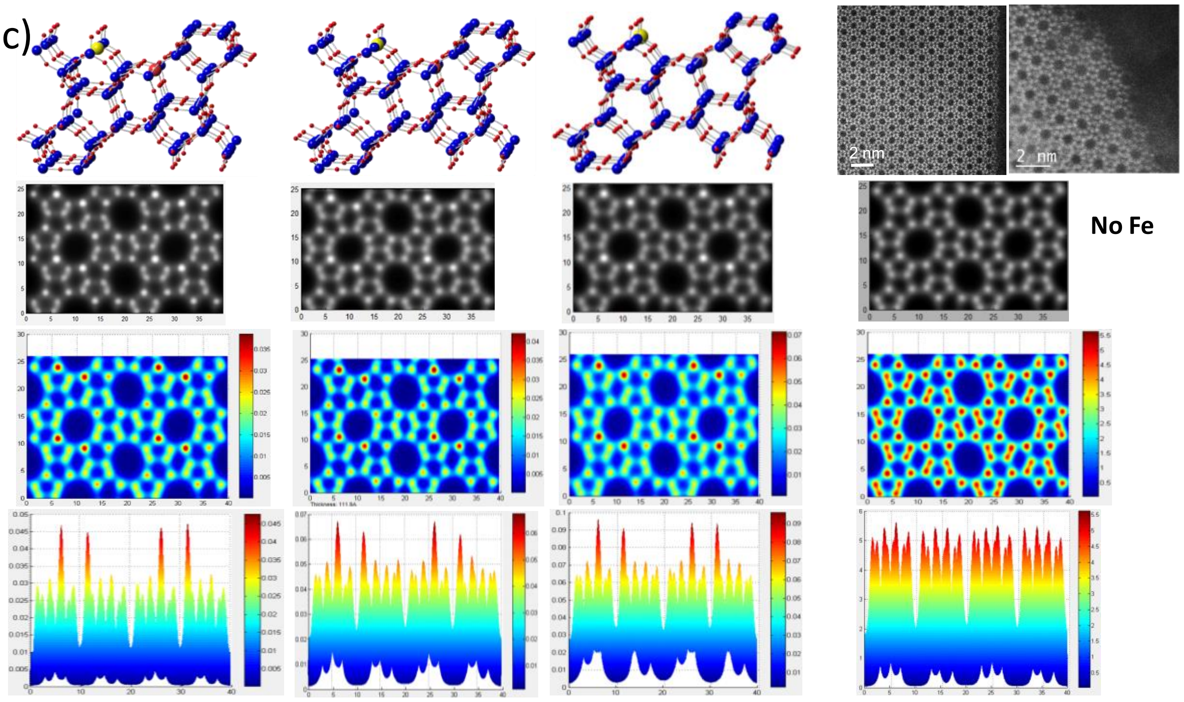

Figure S8. Simulated model and images of C_S-STEM ADF image of Fe-MFI taken with the [010] incidence as a function of specimen thickness and Fe location. a) Schematic unit cell with two single Fe atoms located at T2 and T5 sites corresponding to 2 Fe atoms/unit-cell. b) Effect of the crystal thickness on the signal obtained. c) Image simulations for different position of Fe atoms along *b* axis and the effect on the signal detected. For comparison, two experimental images of Fe free MFI together with the correspondent simulation are also shown.

DFT Calculations

DFT calculations for Na-LTA and Fe-MFI:

DFT calculations for Na-**LTA** were carried out to gain structural insight into $Na_{96}Si_{96}Al_{96}O_{384}$. The starting structure model for dehydrated Na-**LTA** zeolite, with *Fm*-3*c* (#226), $a = b = c = 25.554$ Å, $\alpha = \beta = \gamma = 90°$, was obtained from the International Zeolite Association (IZA) website (Figure S9). Assuming that the resultant model has cubic symmetry, two models with cubic *Pm*-3*n* (#223) symmetry containing 88 Na^+ ions (64 at S6R sites and 24 at 8R sites) were obtained (see Figure S10). It is impossible to distribute 8 Na^+ ions near D4Rs while keeping cubic symmetry of the unit cell (Figure S11); however, 11 distributions with tetragonal symmetry are possible (Figure S12). These 11 4R- Na^+ models were combined with the above-mentioned Models 1 and 2, so that 22 different models were produced (11 for Model 1 and 11 for Model 2). Calculated energies of the two different Na-**LTA** models are summarized in Table S1. Relative energies spanned up to 18 eV. After close inspection of DFT-calculated energies, Model-2-2 was identified as the most stable structure for Na-**LTA**.

(a) Na-**LTA**

A CIF file (Linde_Type_A_Dehydrated.cif) of the Na-**LTA** zeolite ($Na_{96}Si_{96}Al_{96}O_{384}$) was obtained from the International Zeolite Association (IZA) website. The lattice parameters of the unit cell in this CIF file were $a = b = c = 25.554$ Å, and $\alpha = \beta = \gamma = 90°$ (see **Figure S9**).

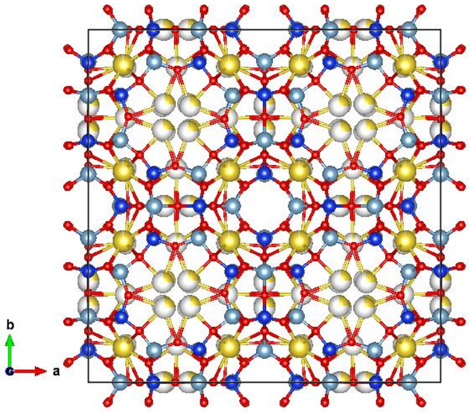

Figure S9. Schematic model along the <100> with the T atoms in blue (light = Al and dark = Si). Na is represented in yellow and O atoms in red.

There are three distinct types of binding sites for Na^+ ions in the **LTA** zeolite, namely, those featuring 6-membered rings (6SR), 8-membered rings (8SR), and double 4-membered rings (D4R) formed by oxygen atoms. Structural models of Na-**LTA** were built via the following two-step procedure. In the first step, only the Na^+ ions residing at the 6MR and 8MR sites were included in the model. There are four possible bindings sites in each of the 8MR sites, and thus there is uncertainty in the positions of Na^+ ions at the 8MR sites. However, by assuming that the resultant model has cubic symmetry, the following two models with cubic (*Pm*-3*n*) symmetry have been obtained. These two models contain 88 Na^+ ions (64 at the 6MR sites and 24 at the 8MR sites, see Figure S10).

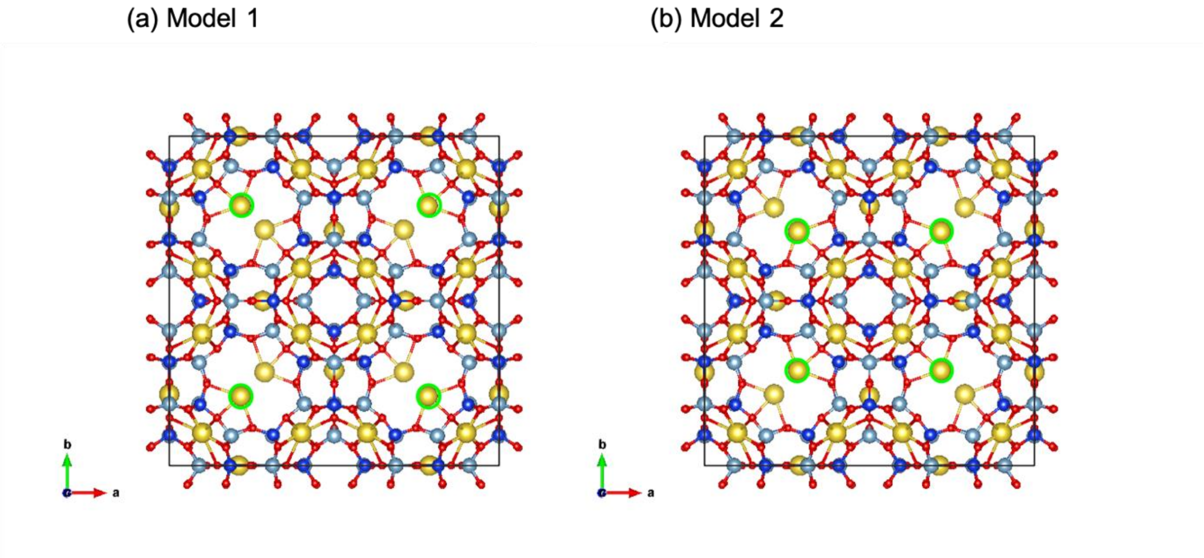

Figure S10. Na-LTA Models. Model 1 a) and Model 2 b) of $Na_{88}Si_{96}Al_{96}O_{384}$. The Na^+ ions at the 8MR sites, which are closest to the reader, are highlighted by light-green circles.

We next considered how to distribute the remaining 8 Na^+ ions at the 4MR sites. As shown in Figure S11, in total, there are 96 4MR sites in the unit cell of Na-**LTA**.

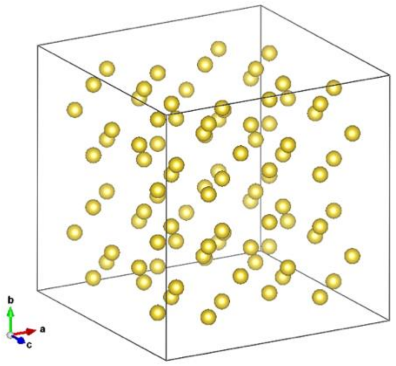

Figure S11. Remaining Na^+. 96 4MR sites in the unit cell of Na-**LTA**.

No matter how 8 Na^+ positions are selected from the 4MR sites, it is impossible for the resultant unit cel of $Na_{96}Si_{96}Al_{96}O_{384}$, or even for the 8 Na^+ ions at the 4MR sites alone, to have cubic symmetry. As such, we considered reasonable positions of Na^+ ions at the 4MR sites. Even though it is not possible to find cubic symmetry for the 8 Na^+ ions, we could obtain the following 11 distributions that satisfy tetoragonal symmetry (Figure S12).

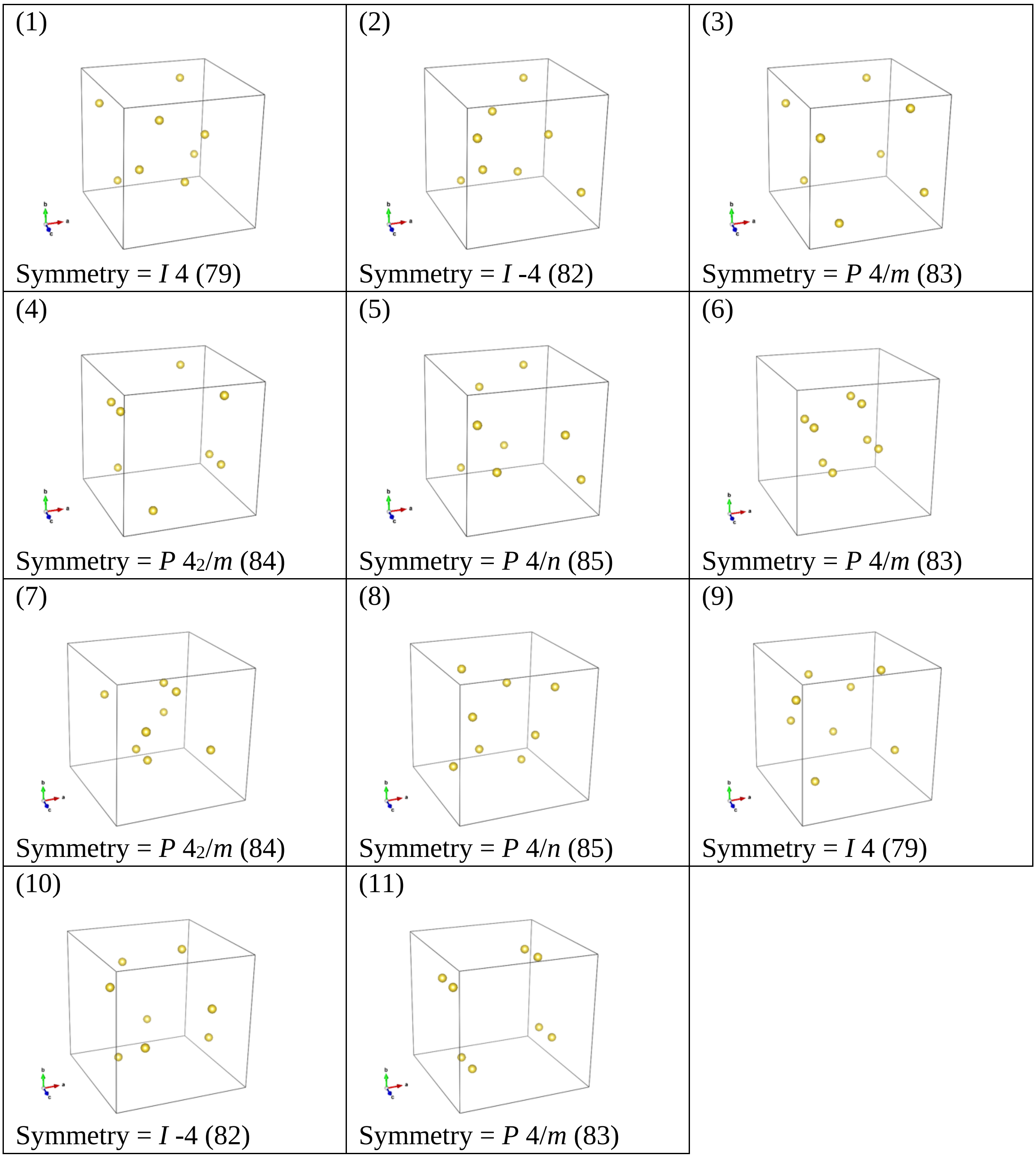

Figure S12. Na^+ distribution. 11 Different distributions of 8 4MR Na^+ ions with tetragonal symmetry.

These 11 4MR Na^+ models were combined with the above-mentioned Models 1 and 2. Consequently, 22 different models in total were produced (11 for Model 1 and 11 for Model 2). In what follows, different models are designated by two numbers; for example, Model 1-2 refers to Model 1 combined with the second 4R-Na^+ model.

We performed periodic density functional theory (DFT) calculations on all these models, using VASP 5.4.1[4]. The projector-augmented wave (PAW) method and the PBE exchange-correlation functional were employed[5], while the kinetic energy cutoff was set to 400 eV. The energy thresholds for self-consistent-field was set to 1×10^{-5} eV. The “LREAL=AUTO” option (for real-space projection), Γ-point-only k-point sampling, and a first-order Methfessel–Paxton

scheme with a σ value of 0.2 were employed for DFT calculations. Because of the large size of the unit cell, geometry optimization was not performed. The reported energies are the energies corresponding to the extrapolated σ→0 limit. VESTA was used to visualize zeolite structures[6].

Calculated energies of the two different Na-**LTA** models are summarized in Table S1. Relative energies spanned up to 18 eV, and the results show that Models 1-9, 1-10, 2-1, and 2-2 are relatively stable. Close inspection further shows that Models 2-1 and 2-2 are more stable than Models 1-10 and 1-11, and Model 2-2 is most stable. DFT calculations thus, suggest that distributions 1, 2, 9, and 10 in Figure S12 have higher populations than others. It should be noted here that by rotating each of these models about an axis parallel to the a, b, and c axes, we can obtain many other equivalent distributions for the Na^+ ions at the 4MR ions. For example, if distribution 1 is rotated by 90 degrees about the axis that is parallel to the *a* axis and passes through (a, b, c) = (0, 1/2, 1/2), we can get another distribution of 8 Na^+ ions with tetragonal symmetry, and many other equivalent distributions can be created. We infer that, although each of these distributions of the 8 Na^+ ions have only tetragonal symmetry, in real unit cells, many such equivalent, yet differently oriented distributions of 4MR Na^+ ions would be adopted, which may finally give the structure solution with cubic symmetry.

Table S1. Energies of 22 different models obtained from single-point energy calculations via Γ-point-only k-point sampling

	E (eV) [a]	ΔE (eV)
Model 1-1	-4701.984248	14.11
Model 1-2	-4702.083728	14.01
Model 1-3	-4698.116060	17.98
Model 1-4	-4705.764721	10.33
Model 1-5	-4706.677485	9.42
Model 1-6	-4698.116066	17.98
Model 1-7	-4705.764727	10.33
Model 1-8	-4706.677493	9.42
Model 1-9	-4715.200540	0.89
Model 1-10	-4715.250721	0.84
Model 1-11	-4709.094663	7.00
Model 2-1	-4715.806152	0.29
Model 2-2	-4716.093674	0.00
Model 2-3	-4709.795376	6.30
Model 2-4	-4706.730313	9.36
Model 2-5	-4707.673397	8.42
Model 2-6	-4709.795409	6.30
Model 2-7	-4706.730326	9.36
Model 2-8	-4707.673555	8.42
Model 2-9	-4703.635672	12.46
Model 2-10	-4703.858052	12.24
Model 2-11	-4699.455112	16.64

[a] Energy (σ→0).

(b) Fe-**MFI**

For computational analyses of **MFI**, the structure of silicalite-1 ($Si_{96}O_{192}$), with *Pnma* (#62), a = 20.0900 Å, b = 19.7380 Å, c = 13.1420 Å, $\alpha = \beta = \gamma = 90°$, was obtained from the International Zeolite Association (IZA) website. Silicalite-1 has 12 crystallographically distinct T-sites at 8(d) positions within the unit cell, and thus 12 models of Fe-substituted **MFI** were built by replacing one of the 8 equivalent sites of each T site by an Fe atom (Figure S13). For the current calculations the oxidation state of Fe was assumed to be +3, which is deemed chemically more plausible than the +4 state[7]. To ensure charge neutrality of the unit cell, one of the 4 neighboring O atoms around the introduced Fe atom was protonated. Consequently, the unit cell of each of the 12 different T-site models consists of 1 Fe atom, 95 Si atoms, 192 O atoms, and one H atom. As each T-site model has 4 possible protonation sites, we examined 48 models in total, table S2. This is in agreement with IR data obtained, which clearly show an OH stretching signal at 3617 cm^{-1} (Al-**MFI** samples show an OH stretching signal at 3610 cm^{-1}) confirming the existence of Si-(OH)-Fe sites (Figure S7 and Figure S14).

The DFT-calculated energies for the models obtained are presented in Table S3. Although some specific sites were seen to have somewhat lower energies than others, small energy differences did not allow us to draw a definitive conclusion on the Fe location at specific sites.

Periodic Kohn–Sham density functional theory (DFT) calculations were performed in the same way as above, but here, geometry optimization was performed. The energy threshold for geometry optimization cycles was set to 1×10^{-4} eV. The difference between the spin-up and spin-down electrons was set to 5. During geometry optimization, atomic positions were relaxed, while cell parameters were held constant.

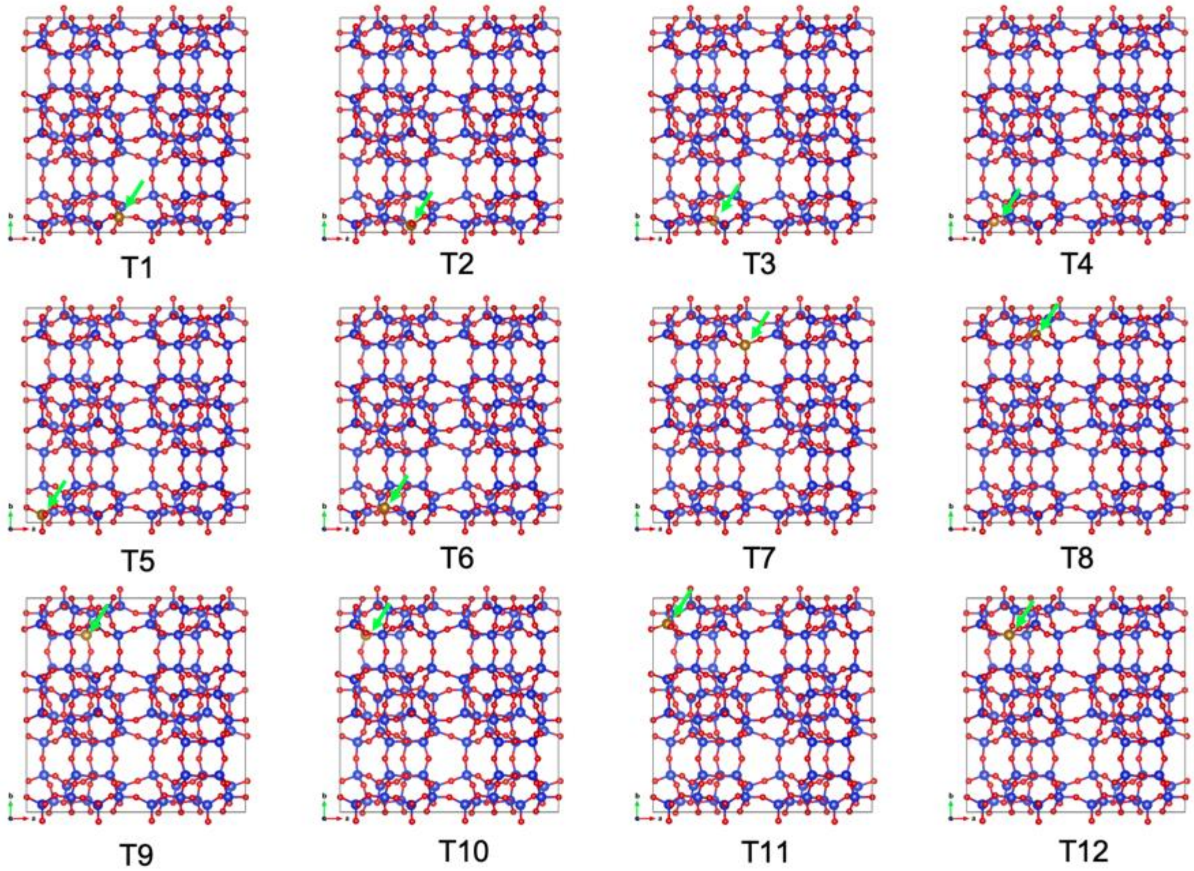

Figure S13. 12 different Fe-substituted MFI models. The light-green arrows indicate the positions of Fe substitution.

Table S2. Initial fractional coordinates of the substituted Si and neighboring O atoms in the T1–T12 models with *P*1 symmetry.

T1	
Si	0.4214 0.0711 0.6898
O_a	0.5012 0.0699 0.7018
O_b	0.3875 0.0743 0.8008
O_c	0.3995 0.1366 0.6251
O_d	0.3972 0.0034 0.6326
T2	
Si	0.3259 0.0336 0.8500
O_a	0.3875 0.0743 0.8008
O_b	0.3290 0.0385 0.9722
O_c	0.3297 0.9555 0.8155
O_d	0.2571 0.0663 0.8106
T3	
Si	0.2792 0.0536 0.0655
O_a	0.3290 0.0385 0.9722
O_b	0.2910 0.1296 0.1060
O_c	0.2034 0.0455 0.0275
O_d	0.2936 0.001 0.1564
T4	
Si	0.1246 0.0514 0.0481
O_a	0.1028 0.9966 0.1326
O_b	0.2034 0.0455 0.0275
O_c	0.1075 0.1265 0.0883
O_d	0.0846 0.0370 0.9443
T5	
Si	0.0721 0.0360 0.8233
O_a	0.0012 0.0699 0.7982
O_b	0.0846 0.0370 0.9443
O_c	0.1301 0.0781 0.7670
O_d	0.0728 0.9589 0.7832
T6	
Si	0.2034 0.0687 0.7197
O_a	0.3297 0.9555 0.8155
O_b	0.2966 0.9545 0.5275
O_c	0.0846 0.0370 0.9443
O_d	0.0728 0.9589 0.7832
T7	
Si	0.4195 0.8274 0.6805
O_a	0.3925 0.8735 0.5883

O_b	0.4911 0.8548 0.7169
O_c	0.3681 0.8311 0.7743
O_d	0.4263 0.7500 0.6431
T8	
Si	0.3152 0.8765 0.8361
O_a	0.3297 0.9555 0.8155
O_b	0.3681 0.8311 0.7743
O_c	0.3217 0.8611 0.9561
O_d	0.2410 0.8582 0.7990
T9	
Si	0.2733 0.8278 0.0402
O_a	0.2799 0.8687 0.1456
O_b	0.3217 0.8611 0.9561
O_c	0.2943 0.7500 0.0579
O_d	0.1976 0.8313 0.0003
T10	
Si	0.1185 0.8279 0.0183
O_a	0.1005 0.8634 0.1251
O_b	0.1976 0.8313 0.0003
O_c	0.0948 0.7500 0.0205
O_d	0.0809 0.8670 0.9275
T11	
Si	0.0657 0.8794 0.8087
O_a	0.0728 0.9589 0.7832
O_b	0.9911 0.8548 0.7831
O_c	0.0809 0.8670 0.9275
O_d	0.1177 0.8370 0.7406
T12	
Si	0.1947 0.8288 0.7092
O_a	0.2090 0.8704 0.6060
O_b	0.2410 0.8582 0.7990
O_c	0.1177 0.8370 0.7406
O_d	0.2116 0.7500 0.6913

Table S3 summarizes the DFT-calculated energies obtained for Fe-**MFI** models. According to our results, the relative energy increases in the order T4 < T7 < T10 < T1 < T8 < T3 < T11 < T5 < T2 < T12 < T6 < T9. Therefore, if the atom displacement process is thermodynamically controlled, preferred positions will have correlations with this order. However, in case the process is kinetically controlled, the DFT-calculated relative energies will not tell which position is likely occupied by Fe. As such, at the current stage, our DFT results only have a qualitative meaning. The low energy of the T4 model is attributed in part to the H-bonding interaction formed between the O–H bond and a nearby O atom (Figure S14). A few authors have reported periodic quantum mechanical calculations on Ti-doped **MFI** zeolites (TS-1). Gale performed periodic DFT calculations on TS-1, and energy differences of different models were small[8]. Pirc and Stare employed periodic Hartree–Fock calculations to compare the

relative stability of different TS-1 models, while imposing symmetry[9]. Here again, energy differences were small. It should be noted that in the case of Fe-substitution, the introduced iron will have a +3 oxidation state.

Table S3. DFT-calculated energies of different models

model	E (eV) [a]	ΔE (eV)	ΔE (kcal/mol)
T1			
O_a	-2280.261447	0.23	5.4
O_b	-2280.340350	0.16	3.6
O_c	-2280.228201	0.27	6.2
O_d	-2280.028826	0.47	10.8
T2			
O_a	-2280.225210	0.27	6.2
O_b	-2280.003738	0.49	11.4
O_c	-2280.077943	0.42	9.6
O_d	-2280.135973	0.36	8.3
T3			
O_a	-2280.283464	0.21	4.9
O_b	-2280.284730	0.21	4.9
O_c	-2280.250880	0.25	5.7
O_d	-2280.134166	0.36	8.3
T4			
O_a	-2280.374685	0.12	2.8
O_b	-2280.333818	0.16	3.7
O_c	-2280.446187	0.05	1.1
O_d	-2280.495999	0.00	0.0
T5			
O_a	-2280.243660	0.25	5.8
O_b	-2280.086159	0.41	9.5
O_c	-2280.194755	0.30	6.9
O_d	-2280.150828	0.35	8.0
T6			
O_a	-2280.073046	0.42	9.8
O_b	-2279.962828	0.53	12.3
O_c	-2280.065084	0.43	9.9
O_d	-2279.889293	0.61	14.0
T7			
O_a	-2280.372733	0.12	2.8
O_b	-2280.385015	0.11	2.6
O_c	-2280.474199	0.02	0.5
O_d	-2280.262022	0.23	5.4
T8			
O_a	-2280.000734	0.50	11.4
O_b	-2280.293268	0.20	4.7

O_c	-2280.032152	0.46	10.7
O_d	-2280.212398	0.28	6.5
T9			
O_a	-2280.049177	0.45	10.3
O_b	-2279.942043	0.55	12.8
O_c	-2279.937853	0.56	12.9
O_d	-2279.936524	0.56	12.9
T10			
O_a	-2280.359118	0.14	3.2
O_b	-2280.289309	0.21	4.8
O_c	-2280.235834	0.26	6.0
O_d	-2280.269318	0.23	5.2
T11			
O_a	-2280.250281	0.25	5.7
O_b	-2280.255905	0.24	5.5
O_c	-2280.263920	0.23	5.4
O_d	-2280.249594	0.25	5.7
T12			
O_a	-2280.138269	0.36	8.2
O_b	-2280.102306	0.39	9.1
O_c	-2280.189695	0.31	7.1
O_d	-2280.170668	0.33	7.5

[a] Energy ($\sigma \rightarrow 0$).

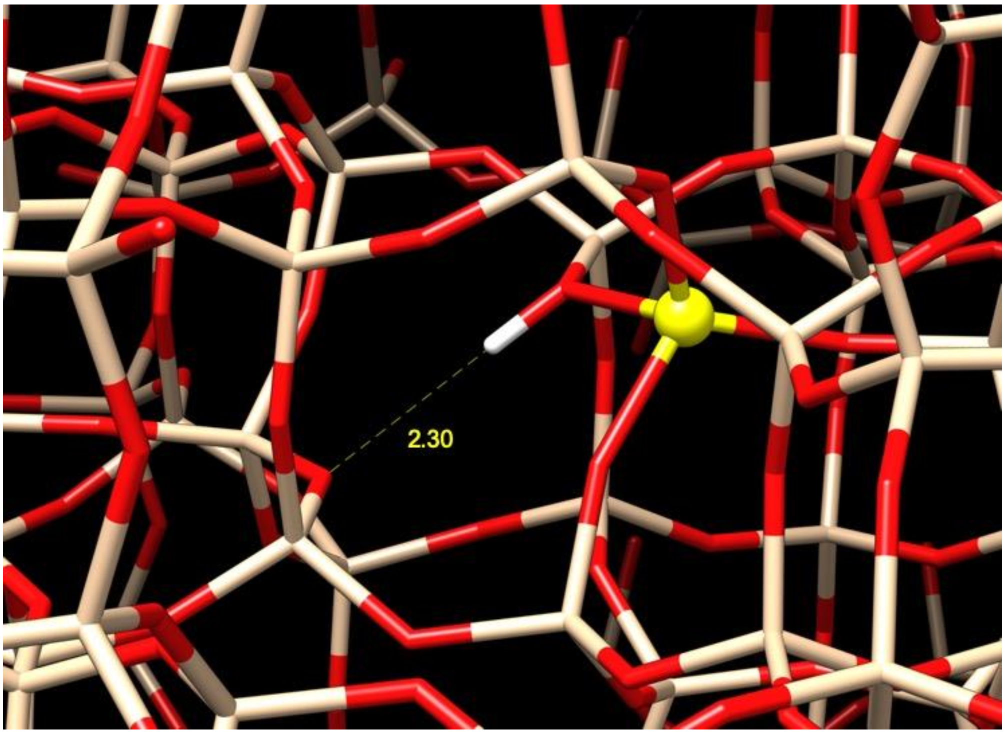

Figure S14. Schematic model of Fe-MFI. Close-up view of the T4(O_d) model with the Fe displayed in yellow and H in white.

Structural study of Na-LTA and Fe-MFI by electron diffraction

LTA: Structure solution was obtained with the highest symmetry $Fm\bar{3}c$ (#226). Modern direct method implemented in *SIR2014* software was used to solve the structure. Chemical formula $(NaSiO_2)_{96}$ was used in the solution process. A reasonable framework structure could be obtained and was consistent with the structure reported by Pluth and Smith. However, considering *hhl* reflection (for example 131) and possibility of Laue class *m*-3, then lower symmetries such as $F\bar{4}3c$ (#219), $F\bar{4}3m$ (# 216) and $Fm\bar{3}$ (# 202) could also give reasonable results. Therefore, the possibilities of space groups with lower symmetry than $Fm\bar{3}c$ are not completely discarded yet. Na (1) and Na (2) in Pluth and Smith reports were detected and located after the refinement, while Na (3) was not observed because of the low occupancy. Atomic coordinates are summarized in Table S4.

Figure S15 corresponds to the three-dimensional electron diffraction tomography (3D-EDT) data and to selected area electron diffraction (SAED) patterns recorded for nano-single crystals of Na-**LTA** in the volume range of (100 ~ 400 nm)^3. The lattice parameters were determined to be a = 25.2 Å, b = 25.0 Å, c = 25.1 Å, α = 89.8˚, β = 89.6˚, γ = 90.3˚ by 3D-EDT. However, no clear evidence of deviation from cubic symmetry were observed considering both accuracy of measurements and small distortion in the electron optics.

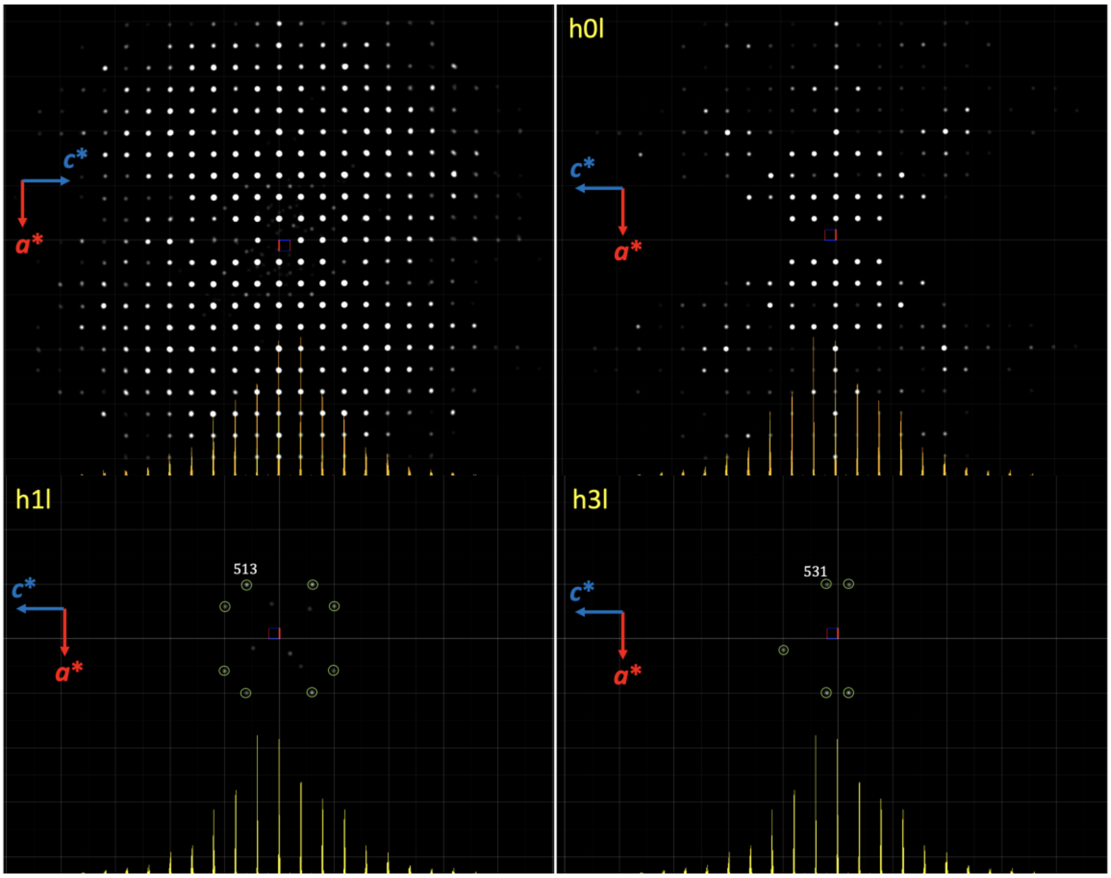

Figure S15. 3D-EDT data. Projections and slice views of the reconstructed 3D-EDT data obtained from a different nanocrystal.

Table S4. Atomic positions of Na-LTA

Atom label	Site	Fractional coordinate *x*	Fractional coordinate *y*	Fractional coordinate *z*
Na (1)		0.10059	0.10059	0.10059
Na (2)		0.20805	0.21554	0
Si		0.18506	0.09339	0
Al		0.18741	0	0.09040
O (1)		0.17038	0.05769	0.05318
O (2)		0.24647	0.11289	0
O (3)		0.14708	0.14505	0

In order to check the possibility that globally averaged symmetry was higher despite local one could be lower, additional EDT data was collected from very small single crystals with dimensions of 100-150 nm. For smaller crystals, the superlattice reflections became much weaker (and close to the noise level) and they were thus hard to distinguish directly in the reconstructed 3D diffraction pattern. However, by comparing the intensity of odd number spots (h, k, l = 2n + 1), (135) reflections are distinct and relatively strong. No other significant violations to the reflection condition of $Fm\bar{3}c$ (No. 226) could be observed, Figure S16.

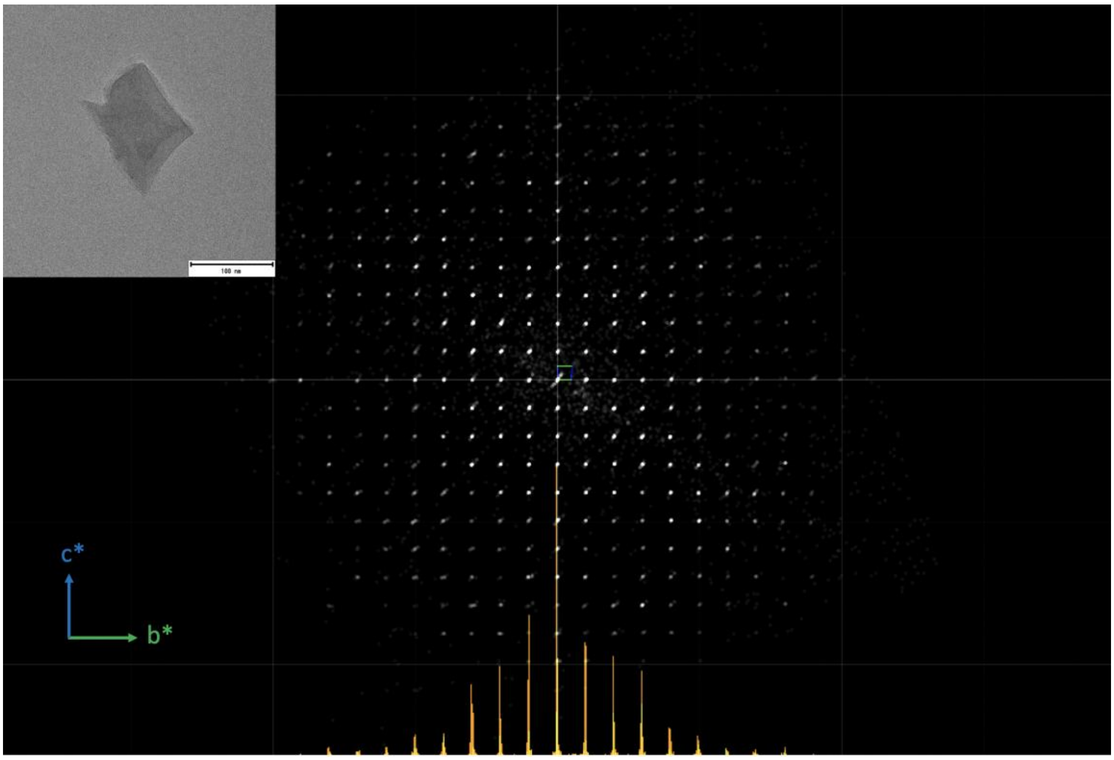

Figure S16. Projection view of the reconstructed 3D-EDT data for a nano crystal.

SAED always yields relatively strong multiple scattering, which may generate extra spots in a diffraction pattern. Nonetheless, when the incidence direction is slightly tilted away from the zone axis, these extra spots caused by multiple scattering should decrease and disappear, Figure S17.

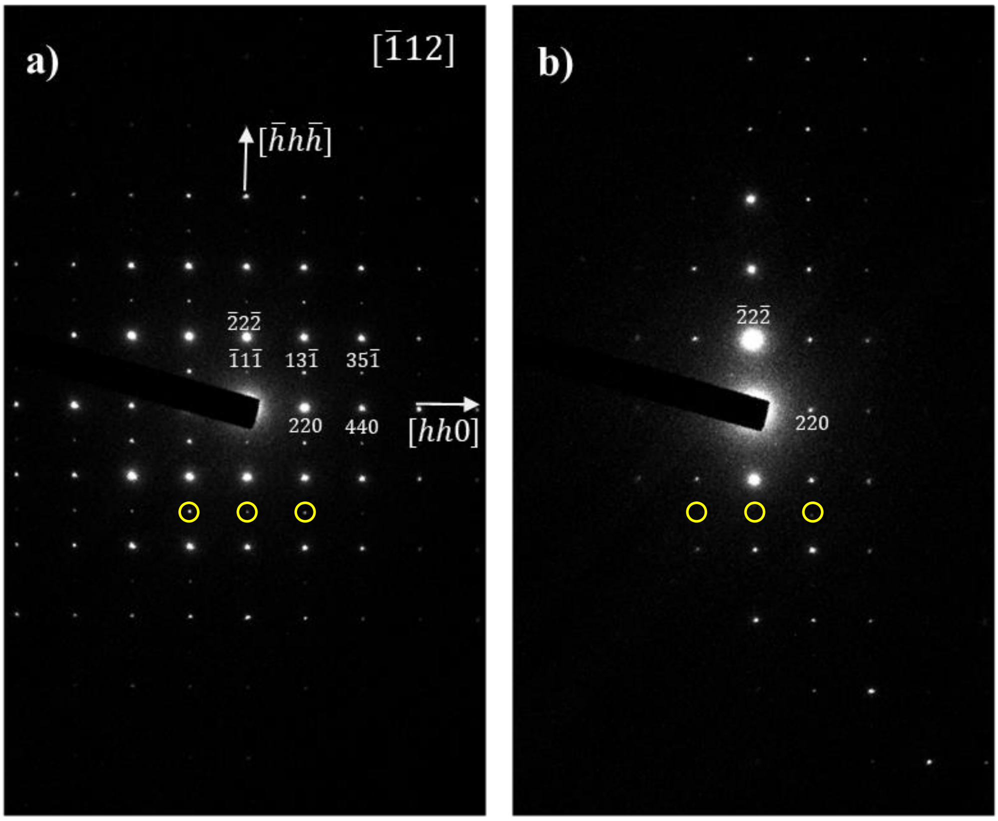

Figure S17. a) SAED pattern at zone axis and b) SAED pattern obtained with an incidence direction that was slightly tilted away from the zone axis. The extra spots are marked by yellow circles

In addition to tilting away from the zone axis, precession electron diffraction (PED) was also used to examine the space group. In the PED patterns, no evidence of violating the reflection rules of $Fm\bar{3}c$ (No. 226) could be found.

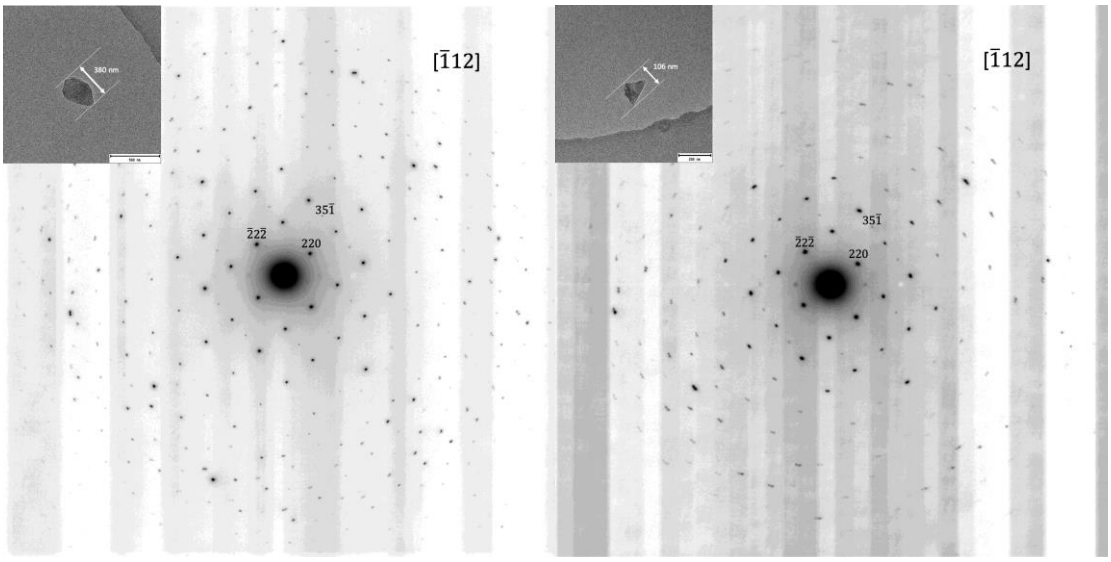

Figure S18. Two sets of PED data for large and nano crystals along the [001] direction.

Fe-**MFI**

The structure of Fe-**MFI** was determined through 3D-EDT, SG *Pnma* with lattice parameters of a = 20.6 Å, b = 20.6 Å, c = 13.9 Å, α = 90.3˚, β = 90.7˚, γ = 90.3˚ from the observed reflections and the extinction rules. The structure was solved and all framework atoms were identified. From the electrostatic potential map, no preferential occupancies of Fe on different T-sites were observed (see also Figure S20). The structure of Fe-MFI was determined by 3D-EDT. From the observed reflections and extinction rules, *Pnma* with lattice parameters: a = 20.6 Å, b = 20.6 Å, c = 13.9 Å, α = 90.3˚, β = 90.7˚, γ = 90.3˚were used for structure solution. There were no preferential occupancies of Fe on different T-sites from the high-quality ED data obtained from rather large volume, in agreement with the STEM analyses.

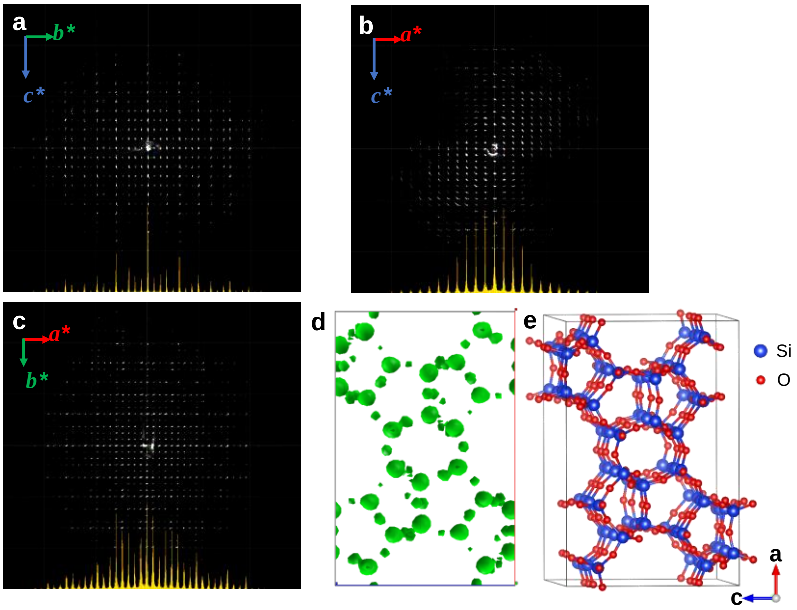

Figure S19. 3D EDT data analyses of Fe-MFI. a) Projection views along [100]. b) [010] and c) [001]. d) Corresponding electrostatic potential distribution. e, Structure solution based on SG: *Pnma* obtained by Sir2014.

References

[1] C. T. Koch, Arizona State University **2002**.
[2] S. Hovmöller, *Ultramicroscopy* **1992**, *41*, 121-135.
[3] J. J. Pluth, J. V. Smith, *J. Am. Chem. Soc.* **1980**, *102*, 4704–4708.
[4] G. Kresse, J. Furthmuller, *Phys. Rev. B* **1996**, *54*, 11169-11186.
[5] J. P. Perdew, K. Burke, M. Ernzerhof, *Phys. Rev. Lett.* **1996**, *77*, 3865-3868.
[6] K. Momma, F. Izumi, *J. Appl. Crystallogr.* **2011**, *44*, 1272-1276.
[7] H. Y. Chen, W. M. H. Sachtler, *Catal. Today* **1998**, *42*, 73-83.
[8] J. D. Gale, *Solid State Sciences* **2006**, *8*, 234-240.
[9] G. Pirc, J. Stare, *Acta Chim. Slov.* **2008**, *55*, 951-959.
